# Single-cell transcriptomic analysis reveals a systemic immune dysregulation in COVID-19-associated pediatric encephalopathy

**DOI:** 10.1038/s41392-023-01641-y

**Published:** 2023-10-18

**Authors:** Yi Wang, Laurence Don Wai Luu, Shuang Liu, Xiong Zhu, Siyuan Huang, Fang Li, Xiaolan Huang, Linying Guo, Jin Zhang, Haiyan Ge, Yuanyuan Sun, Yi Hui, Yanning Qu, Huicong Wang, Xiaoxia Wang, Weilan Na, Juan Zhou, Dong Qu, Jun Tai

**Affiliations:** 1 Experimental Research Center, Capital Institute of Pediatrics, Beijing, 100020 P.R. China; 2 School of Life Sciences, University of Technology Sydney, Sydney, Australia; 3 Department of Critical Medicine, Children’s Hospital Affiliated Capital Institute of Pediatrics, Beijing, 100020 P.R. China; 4 Central & Clinical Laboratory of Sanya People’s Hospital, Sanya, Hainan 572000 P. R. China; 5 Department of Otorhinolaryngology Head and Neck Surgery, Children’s Hospital Affiliated Capital Institute of Pediatrics, Beijing, 100020 P.R. China

**Keywords:** Infectious diseases, Infection

## Abstract

Unraveling the molecular mechanisms for COVID-19-associated encephalopathy and its immunopathology is crucial for developing effective treatments. Here, we utilized single-cell transcriptomic analysis and integrated clinical observations and laboratory examination to dissect the host immune responses and reveal pathological mechanisms in COVID-19-associated pediatric encephalopathy. We found that lymphopenia was a prominent characteristic of immune perturbation in COVID-19 patients with encephalopathy, especially those with acute necrotizing encephalopathy (AE). This was characterized a marked reduction of various lymphocytes (e.g., CD8^+^ T and CD4^+^ T cells) and significant increases in other inflammatory cells (e.g., monocytes). Further analysis revealed activation of multiple cell apoptosis pathways (e.g., granzyme/perforin-, *FAS-* and *TNF*-induced apoptosis) may be responsible for lymphopenia. A systemic *S100A12* upregulation, primarily from classical monocytes, may have contributed to cytokine storms in patients with AE. A dysregulated type I interferon (IFN) response was observed which may have further exacerbated the *S100A12*-driven inflammation in patients with AE. In COVID-19 patients with AE, myeloid cells (e.g., monocytic myeloid-derived suppressor cells) were the likely contributors to immune paralysis. Finally, the immune landscape in COVID-19 patients with encephalopathy, especially for AE, were also characterized by NK and T cells with widespread exhaustion, higher cytotoxic scores and inflammatory response as well as a dysregulated B cell-mediated humoral immune response. Taken together, this comprehensive data provides a detailed resource for elucidating immunopathogenesis and will aid development of effective COVID-19-associated pediatric encephalopathy treatments, especially for those with AE.

## Introduction

SARS-CoV-2 (severe acute respiratory syndrome coronavirus-2) which causes COVID-19 (coronavirus disease 2019), is still a serious global public health problem.^1,2^ The ongoing global pandemic of COVID-19 has caused >630 million cumulative cases, resulting in over 6.60 million deaths.^2,3^ Fever, cough, shortness of breath and sore throat are common COVID-19 clinical manifestations.^4^ Neurological symptoms are also common including ageusia, anosmia, myalgia and headaches.^5^ Lately, many studies have reported severe COVID-19-associated neurological complications, including encephalopathy, encephalitis, Guillain Barre syndrome, stroke and skeletal muscle involvement, which increases the risk of death.^6^ Herein, elucidating the pathological features and mechanisms of severe COVID-19-related neurological manifestations is critical for bettering the disease outcomes.

Encephalopathy is a severe SARS-CoV-2-related CNS (central nervous system) complication, characterized by diffuse brain dysfunction.^7^ COVID-19-associated encephalopathy typically have altered consciousness, ranging from delirium, confusion to deep coma. Delirium, which manifests as an acutely developed fluctuation disturbance in awareness and attention, is commonly observed as a typical feature of mild to moderate encephalopathy in COVID-19 patients.^7^ There may be other clinical manifestations such as headache, seizures, or extrapyramidal signs. In particular, COVID-19 patients with encephalopathy are critically ill, with the majority of patients requiring mechanical ventilation. Furthermore, COVID-19 patients with encephalopathy usually suffer from multiple organ failure which includes respiratory, circulatory, renal and/or hepatic failure, sepsis, shock and coagulopathies.^7^ Therefore, understanding the immune response and pathophysiology of COVID-19 related encephalopathy is critically important to developing effective therapies.

There have been multiple mechanisms hypothesized for COVID-19-related encephalopathy including SARS-CoV-2-associated host response and coagulopathies-related cerebral hypoxic-ischemic injury.^8^ Recently, many studies have reported that the inflammatory cytokine storm in COVID-19 is triggered by an intense inflammatory response against SARS-CoV-2 virus, which is featured by the overproduction of chemokines (e.g., CCL2, CCL3, CCL5, ect.) and proinflammatory cytokines (e.g., IL-1β, IL6, IL8, MIP, etc.).^9,10^ This results in metabolic dysfunction/hypoxia and subsequently leads to acute respiratory distress syndrome (ARDS), multiple organ failure^11–13^ and are thought to be responsible for diffuse brain dysfunction, contributing to metabolic/hypoxic encephalopathy.^14^ In agreement, several proinflammatory cytokines, including interleukin (IL)-8, IL-15, IL-6, MIP-1b (macrophage inflammatory protein-1b) and TNF-α, were significantly elevated in COVID-19 patients with encephalopathy and was correlated with its severity.^13^ The elevated levels of both TNF-α and IL-8 promote the secretion of von Willebrand factor, an indicator of endothelial injury that is found to be increased in COVID-19 patients with encephalopathy.^10^ The increased concentrations of IL-6 suppress cleavage of von Willebrand factor resulting in the accumulation of multimers that actively facilitate platelet aggregation.^15^ In addition, the elevated levels of several proinflammatory cytokines such as IL-8, IL-15, IL-6 and MIP-1b were detected in a subgroup of COVID-19 patients with encephalopathy and exhibited a correlation with BBB (blood-brain barrier) breakdown.^13^ Although recent evidence supports the existence of aberrant immune responses in COVID-19 patients with encephalopathy, a comprehensive understanding of the immune landscape is still required to clarify the potential changes related to encephalopathy and elucidate the pathogenic mechanisms in COVID-19-associated encephalopathy.

scRNA-seq (Single-cell RNA sequencing) is a powerful tool for elucidating immune response and has been used for COVD-19 studies.^16,17^ Although the current scRNA-seq studies of COVID-19 have offered crucial molecular and cellular insights, a comprehensive immune landscape is still needed to uncover the specific pathological hallmarks and mechanisms underlying COVID-19-associated encephalopathy. In this study, we conducted peripheral blood mononuclear cell (PBMC) scRNA-seq on a cohort of 17 pediatric participants, including COVID-19 patients with or without encephalopathy, convalescent COVID-19 encephalopathy patients, and healthy donors in order to provide a comprehensive and detailed transcriptomic landscape of the immune response in pediatric patients with COVID-19-associated encephalopathy. This will facilitate a greater understanding of the underlying pathogenic immune response and aid improved outcomes for COVID-19 associated encephalopathy.

## Results

### Single-cell PBMC transcriptomic profiling of COVID-19 patients with encephalopathy

**Fig. 1:**
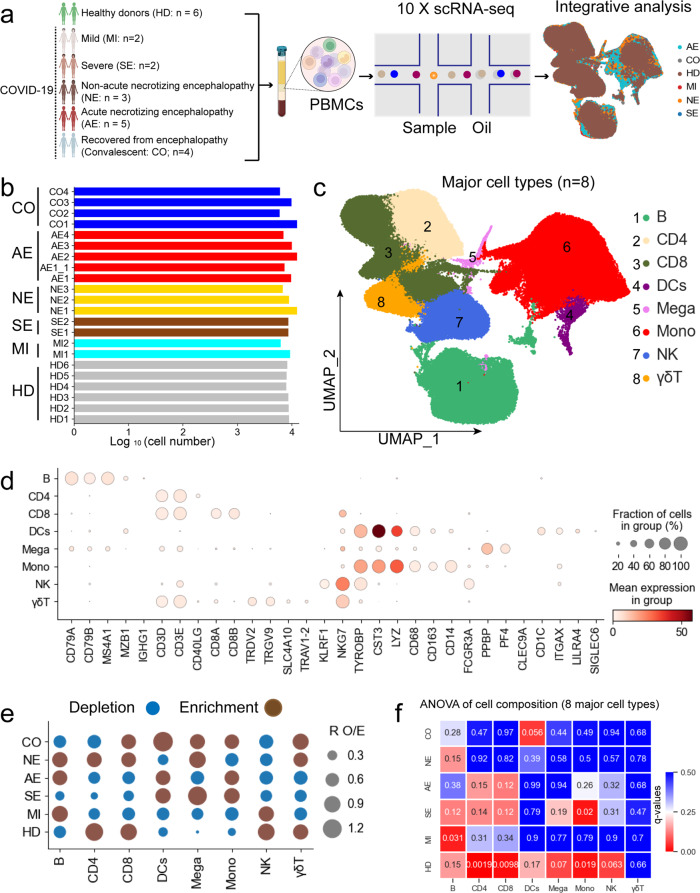
PBMC single-cell transcriptomic study design and overview of results. **a** Diagram depicting the overall study design. 22 samples were collected from 17 individuals, including 6 healthy donors and 11 COVID-19 patients (2 patients with mild-moderate disease, 2 patients with severe disease, 3 patients with non-acute necrotizing encephalopathy and 4 patients with acute necrotizing encephalopathy). **b** Box plots illustrating the log_10_ transformed number of cells for each sample. 6 HD samples were obtained from healthy donors, 2 MI samples from patients with mild-moderate symptoms, 2 SE samples from patients with severe symptoms, 3 NE samples from patients with non-acute necrotizing encephalopathy, 5 AE samples from patients with acute necrotizing encephalopathy and 4 CO samples from patients who recovered from encephalopathy (convalescent). **c** The clustering result (Left row) of 8 major cell types (right row) from 22 samples. Each point represents one single cell, colored according to cell type. **d** Dot plots of the 8 major cell types (Columns) and their marker genes (Rows). **e** Disease preference of major cell clusters as estimated using R_O/E_. **f** Heatmap showing the association between cell composition and disease types. The color represents ANOVA *q*-values

To elucidate the immune features of COVID-19-associated encephalopathy, we performed scRNA-seq on 22 PBMC samples from 17 pediatric patients to investigate the immunological and pathogenic mechanisms in COVID-19 patients with encephalopathy. This included 7 hospitalized patients with encephalopathy (4 with acute necrotizing encephalopathy (AE) and 3 with non-acute necrotizing encephalopathy (NE)), 2 hospitalized patients with severe symptoms but no encephalopathy (SE), 2 patients with mild or moderate symptoms (MI), as well as 6 healthy donors (HD) (Fig. 1, Supplementary Fig. 1a and Supplementary Table 1). The clinical and laboratory notes for COVID-19 patients in this study are detailed in Supplementary Table 1. Mild and moderate COVID-19 patients were combined into one group. According to disease types and severity, the 22 samples were classified into 6 conditions: AE (*n* = 5), NE (*n* = 3), MI (*n* = 2), SE (*n* = 2), CO (convalescent; *n* = 4, of whom 1 was paired with AE case, 3 were paired with NE case) and HD (*n* = 6) (Fig. 1a and Supplementary Fig. 1a). We compared the cellular and molecular mechanisms underlying differences between encephalopathy and non-encephalopathy cases as well as between AE and NE cases.

We sequenced 195,235 cells from 22 samples (Fig. 1b) with 178,435 cells remaining after QC. On average, there were 4056 unique molecular identifiers (UMIs), which represented 1700 genes (Fig. 1b and Supplementary Fig. 1b–e). After correction for mitochondrial read counts, read depth and data integration with PCA (principal component analysis) into an unbatched and comparable dataset (Supplementary Fig. 1b–e), 40,803 cells (22.86%) were from the AE group, 25,862 cells (14.49%) were from the NE group, 14,573 cells (8.16%) were from the MI group, 16,408 cells (9.22%) were from SE group, 32,639 cells (18.29%) were from the CO group and 48,150 cells (26.98%) were from the HD group.

**Fig. 2:**
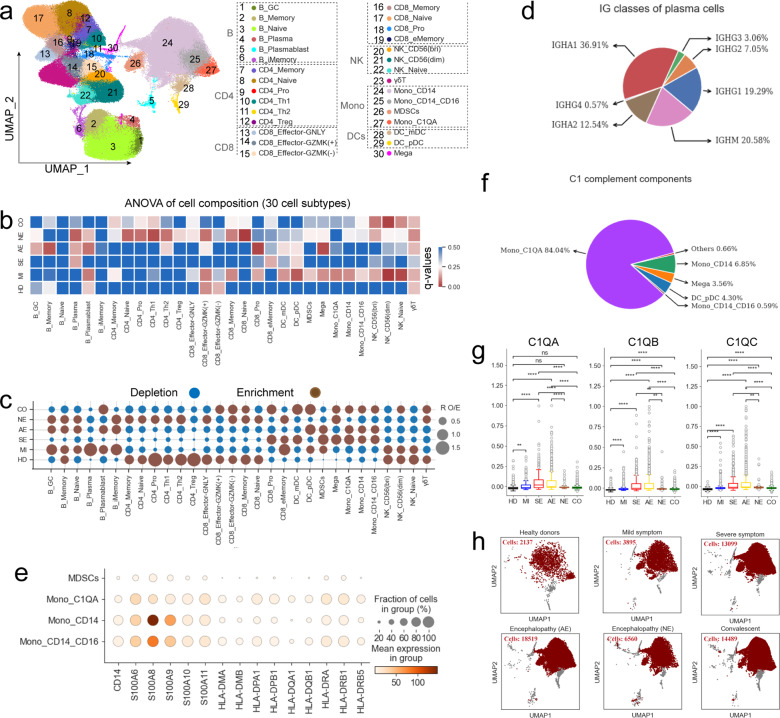
Associations between COVID-19 disease severity and PBMC cellular composition. **a** UMAP projection showing the 30 cellular subtypes identified from 22 samples. Each dot depicts a single cell while color represents the cell subtype. **b** Heatmap showing the *p*-values from ANOVA analysis of differences in cell subtype composition between disease types. Disease severity: HD, MI, SE, AE, NE and CO. **c** Dot plot depicting the 30 cell subtype disease preference as calculated using R_O/E_. **d** Classes of heavy chains for plasma cells from AE. **e** Dot plot showing the expression of selected monocyte marker genes in monocyte subtypes. **f** Pie chart depicting the relative contribution of each cell subtype to the C1 complement components. **g** Box plots showing C1QA, C1QB, and C1QC expression in Mono_ C1QA cells between different groups. Significant differences were determined with a two-sided Student’s T-test with Bonferroni correction. Standard Error (SE) and median are shown. **h** UMAP projection density plots of Mono_CD14 cells from different groups

To investigate immune cell populations in COVID-19 patients with encephalopathy, we identified eight major cell types and 30 cell sub-types according to canonical marker gene expression and uniform manifold approximation and projection (UMAP) clustering (Fig. 1c,d, Fig. 2a and Supplementary Table 2). Most cell types/subtypes were observed across several COVID-19 patients which suggests common immune features among COVID-19 patients. Therefore, we successfully defined the cell population/subpopulation composition in peripheral blood (Figs. 1, 2, Supplementary Fig. 1, 2 and Supplementary Table 2).

Clear differences were seen in the UMAP projection (Fig. 1a). The disease preferences for major cell types were illustrated using R_O/E_^9^ (Fig. 1e). Notably, most lymphocyte populations (including CD8^+^ T, CD4^+^ T, NK, γδT cells) were significantly reduced in COVID-19 with acute necrotizing encephalopathy group (Fig. 1e and Supplementary Fig. 2e), suggesting lymphopenia may be a prominent characteristic COVID-19 associated acute necrotizing encephalopathy. In contrast, most myeloid cells (e.g., monocytes) in acute necrotizing encephalopathy samples were enriched (Fig. 1e). The relationship between lymphopenia, increased myeloid cells and acute necrotizing encephalopathy were also associated when analyzed with ANOVA (Fig. 1f), and were not reversed when patients recovered (Supplementary Fig. 2e). These results (lymphopenia and elevated myeloid cells) were also seen in COVID-19 patients displaying severe disease (Fig. 1e and Supplementary Fig. 2), consistent with previous reports.^9,18^ In addition, we found that the compositional change in peripheral immune cells from COVID-19 non-acute necrotizing encephalopathy patients was similar to patients with mild/moderate symptoms (Fig. 1e and Supplementary Fig. 2), and their cell composition characteristics have been defined by previous reports.^9,16^ Taken together, we highlight lymphopenia and increased myeloid cells as prominent features in COVID-19 encephalopathy patients.

### Association between PBMC composition and disease type

We further dissected the changes in composition for different cell subtypes and their association to disease type. We used ANOVA (analysis of variance) to assess this association based on six disease conditions and significant associations were identified (Fig. 2b). Strikingly, most B cell subtypes appeared to be associated with all disease types, while B_Plasma and B_Plasmablast showing an obvious association with COVID-19 patients with encephalopathy (Fig. 2b). *CD38*, *MZB1*, *MKI67*, *JCHAIN*, *XPB1* and *PRDM1* were highly expressed in B_Plasmablast (Supplementary Fig. 1f-g and Supplementary Table 2), confirming this cell subtype as cycling plasma cell (also known as plasmablast cell). B_Plasma highly expressed *CD38*, *MZB1* and *IGHM*, but not for *MKI67*, demonstrating this subcluster as plasma cells. We found that plasma and cycling plasma cells were enriched in the COVID-19 group with encephalopathy compared to healthy donors (Fig. 2c). Further work confirmed that the percentage of plasma and cycling plasma cells were elevated in the COVID-19 group with encephalopathy, and the former was significant (Supplementary Fig. 3a), suggesting that elevated plasma cells provide protective neutralizing antibodies against SARS-CoV-2. In particular, the immunoglobulin constant region genes for IgM, IgG1, IgG2, IgA1 or IgA2 were highly expressed in plasma cells, providing evidence for secretion of antigen-specific antibodies by these cells. These observations imply that, similar with previous findings in COVID-19 patients,^19,20^ the serum of these patients with encephalopathy may also have high SARS-CoV-2 antibody levels.

The increase of cycling plasma cells appears to be derived from B_Memory cells according to pseudo-time analysis (Supplementary Fig. 3b). The B_Memory (as the memory B cell cluster), featured by *CD27*, *AIM2*, *COCH* and *GRP183* (Supplementary Fig. 1 and Supplementary Table 2), was the unique source of plasmablast B cells. However, the elevation of plasma cells appears to originate from B_iMemory (an intermediate transition memory B subtype) and B_GC (a germinal center B subtype) clusters (Supplementary Fig. 3b), consistent with previous finding.^9^ Transition from B_imemory/B_GB clusters to B_Plasma was validated by the PAGA (Partition-based graph abstraction) map, because we found strong connectivity between B_imemory/B_GC and B_Plasma (Supplementary Fig. 3b). Interestingly, we also found that B_imemory and B_GC clusters were enriched in COVID-19 patients with encephalopathy, showing an association with encephalopathy (Fig. 2b, c). This data revealed the association between B cell subsets and COVID-19-related disease.

**Fig. 3:**
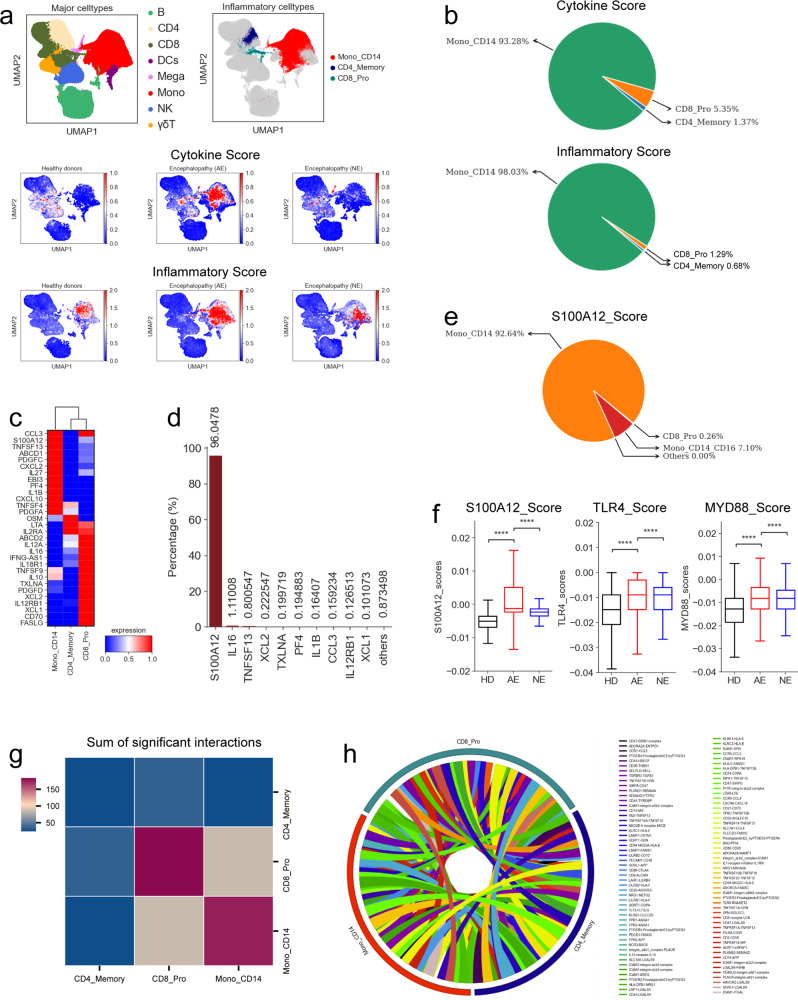
Contribution of *S100A12* to COVID-19 cytokine storms in severe disease. **a** UMAP projections of PBMCs. Colored based on the 8 major cell types (top left), 3 hyper-inflammatory cell subtypes (top right), cytokine (Middle) and inflammatory score (Bottom). **b** Pie charts depicting the relative contribution of each inflammatory cell subtype to the cytokine and inflammatory scores. **c** Heatmap depicting the expression of cytokines within each hyper-inflammatory cell subtype identified. **d** Bar chart depicting the relative contribution of the top 10 cytokines in COVID-19 patients with acute necrotizing encephalopathy. **e** Pie charts depicting the relative contribution of each cell subtype to the *S100A12*-score. **f** Box plots depicting the S100A12, TLR4 and MYD88 gene expression scores between different groups. Significant differences were determined with a two-sided Student’s *T*-test with Bonferroni correction. Standard Error (SE) and median are shown. **g** Heatmap of the sum of significant interaction among the 3 hyper-inflammatory cell subtypes. **h** Circos plot depicting the ligand-receptor pair interactions between the 3 hyper-inflammatory cell subtypes

For T cells, our analysis found that most T cell subtypes were associated with encephalopathy recovery status, while two proliferative T cells subsets (CD4_Pro and CD8_Pro) displayed distinct associations with COVID-19 encephalopathy types (Fig. 2b). CD4_Pro cell cluster, which expresses *MKI67*, *CCR7* and *TCF7* at relatively high levels (Supplementary Fig. 1 and Supplementary Table 2), were derived from naïve CD4 cells (CD4_Naïve) based on the PAGA map (Supplementary Fig. 3c). Although CD4_Pro were decreased in COVID-19 patients (Fig. 1c and Supplementary Fig. 3d), this cluster exhibited an association with non-acute necrotizing encephalopathy (Fig. 2b). Specially, CD4_Pro seemed to be an intermediate state (Fig. 3c), exhibiting high connectivity between naïve CD4 and regulatory T cells (CD4_Treg). Likewise, CD4_Naïve cluster were also reduced in COVID-19 patients, especially for the encephalopathy and severe groups (Fig. 2c and Supplementary Fig. 3d). The decrease in proliferative CD4 and their precursor CD4_Naïve T cells may partially suggest a dysregulated adaptive immune response in COVID-19, especially for those patients displaying encephalopathy.

CD8_Pro, a proliferative effector memory CD8^+^ T cell subset with proliferative, cytotoxic and memory markers (e.g., *MKI67*, *TYMS*, *GPR183*, *S100A4*, *GNLY*, *GZMK*, etc.) (Supplementary Fig. 1 and Supplementary Table 2), was significantly increased in the groups with severe disease and acute necrotizing encephalopathy, particularly in the convalescence stage (Fig. 2c). Specially, CD8_Pro had an obvious association with COVID-19 acute necrotizing encephalopathy patients (Fig. 2b). According to the PAGA analysis, CD8_Pro appeared to be derived from memory CD8^+^ T cells (CD8_Memory) (Supplementary Fig. 3e), which expresses *GPR183*, *S100A4*, *CD27*, *CCL5* and *GZMA* at relatively high levels (Supplementary Fig. 1 and Supplementary Table 2). The PAGA map revealed that the CD8_Memory subset was an intermediate state, connecting the naïve subset (CD8_Naïve) to most other CD8 T cell subtypes (Supplementary Fig. 3e). Though not significant, we observed that the CD8_Memory subset was reduced in PBMCs from COVID-19 patients, especially for the severe disease and acute necrotizing encephalopathy groups (Fig. 2c and Supplementary Fig. 3f). The increase in CD8_Pro and reduction in their precursor CD8_Memory cells might partially indicate stronger CD8^+^ T cell responses in COVID-19 groups with severe disease and encephalopathy patients, resulting in potential pathogenic injury.

Compared to proliferative CD8^+^T cells which were increased in COVID-19 patients, most T and NK cell subsets (e.g., CD4_Treg, CD4_Th1, CD4_Th2, γδT, NK_CD56^(dim)^) decreased, especially for those displaying severe disease and acute necrotizing encephalopathy. Their association with different disease types were also varied (Fig. 2b, c). Decreased γδT cells has been previously reported in the PBMCs from severe COVID-19 patients.^9,21^

Unlike the lymphocytes which were depleted in COVD-19 patients with acute necrotizing encephalopathy, most myeloid cell subsets (e.g., Mono_CD14) were increased in peripheral blood (Fig. 2c), consistent with previous results in COVID-19 patients with severe symptoms.^9,16^ Different myeloid cell subsets exhibited different associations with COVID-19 disease types (Fig. 2b), i.e. megakaryocytes had a significant association with COVID-19-associated acute necrotizing encephalopathy. Among myeloid cells, we identified a specific subtype in peripheral blood (Fig. 2a, Supplementary Fig. 1), known as myeloid-derived suppressor cells (MDSCs), which suppresses T cell response and are increased in inflammatory conditions.^22,23^ The PAGA analysis confirmed that MDSCs was derived from Mono_CD14, confirming this cluster was monocytic MDSCs (Supplementary Fig. 3f). In peripheral blood, in addition to high expression of CD14, this cluster has the phenotype HLA-DR^-/lo^, consistent with previous observations that monocytic MDSCs had a downregulation of HLA-II molecules (Fig. 2e).^24^ Particularly, upregulation of calprotectin (*S100A8/A9*), and immunosuppressive functions (e.g., the elevated *PD-L1* expression and the decreased *CD62L* expression) are reported hallmarks of monocytic MDSCs. In line with previous reports,^24,25^ the monocytic MSDCs had relatively high expression of calprotectin (e.g., *S100A8* and *S100A9*) and *PDL-1* (especially for HLA-DR molecules) as well as low expression of *CD62L* (Fig. 2e and Supplementary Fig. 3i). Monocytic MSDCs were also higher in COVID-19 patients, especially for severe disease and acute necrotizing encephalopathy (Supplementary Fig. 3h). These data suggested that this cluster in COVID-19 patients with AE has highly resembled MDSCs.

In addition to monocytic MDSCs, C1QA/B/C-expressing monocytes (Mono_C1QA) were also identified (Fig. 2a and Supplementary Fig. 1), which were more enriched in patients with acute necrotizing encephalopathy and severe symptom (Supplementary Fig. 2h). Further analysis indicated that Mono_C1QA was the major source of C1 complement components (Fig. 2f and Supplementary Fig. 3j). Genes encoding complement components, including *C1QA*, *C1QB* and *C1QC*, were significantly upregulated in COVID-19 severe disease and acute necrotizing encephalopathy patients compared to healthy donors and other disease types (Fig. 2g). This suggests that *C1QA*, *C1QB* and *C1QC* may be valuable for predicting disease severity and diagnosis of COVID-19 associated acute necrotizing encephalopathy. In addition, differential UMAP projection patterns for monocytes in different COVID-19 disease conditions suggests a perturbed transcriptome hallmark in these cells (Fig. 2h).

### *S100A12*, mainly by classical monocytes, contributed to cytokine storms in COVID-19 acute necrotizing encephalopathy patients

Cytokine storms are a primary contributor to multiple organ failure and death for COVID-19. The hallmarks of cytokine storms for severe COVID-19 disease have been dissected but less is known about its characteristics in COVID-19-associated encephalopathy. Here, we investigated the potential sources of cytokine release. A cytokine and inflammatory score was defined for each cell cluster based on the expression of predefined cytokine and inflammatory genes, respectively, (Supplementary Table 3). These two interrelated scores were then used as indicators for assessing the contribution for each cell cluster to inflammatory cytokine storm. A significant upregulation of cytokine and inflammatory genes were observed in COVID-19 acute necrotizing encephalopathy patients (Fig. 3a and Supplementary Fig. 4a, b). This suggests that an inflammatory cytokine storm was present in these patients. Fourteen subsets, including three CD4 T cell subtypes (CD4_Memory, CD4_Th1 and CD4_Th2), five CD8 T cell subtypes (CD8_Effectory-GNLY, CD8_Effectory-GZMK^(+)^, CD8_Effectory-GZMK^(-)^, CD8_Pro and CD8_Memory), three NK cell subtypes (NK_CD56 ^(dim)^, NK_CD56^(bri)^ and NK_Naïve), two myeloid cell subtypes (Mega and Mono_CD14) and one γδT cell subtype, were detected with significant inflammatory and cytokine scores increased (Supplementary Fig. 4c). This suggests that these subtypes might be key contributors to inflammatory cytokine storms. Further analysis found that only three cell subtypes (Mono_CD14, CD8_Pro and CD4_Memory) were observed with significantly elevated cytokine scores in COVID-19 acute necrotizing encephalopathy patients compared to healthy donors and those with non-acute necrotizing encephalopathy (Supplementary Fig. 4d). This suggests that these subtypes are the main contributors to cytokine storm. Interestingly, CD14-expressing monocytes (Mono_CD14), which were the largest contributors of inflammatory cytokine storm in COVID-19 acute necrotizing encephalopathy patients (Fig. 3b), have been validated as critical sources of cytokine storms in patients with severe COVID-19 disease in a previous study.^9^

Next, we determined the proportion of these three cell subsets in COVID-19 patients with encephalopathy. We observed that these subsets were significantly elevated in COVID-19 acute necrotizing encephalopathy patients (Supplementary Fig. 4e) and that the proportion of inflammatory cells displayed different enrichment patterns (Fig. 2c). Additionally, we evaluated their inflammatory gene signatures in COVID-19 acute necrotizing encephalopathy patients. In each inflammatory cell subset, unique expression patterns for pro-inflammatory cytokine genes were observed such as *S100A12*, *CCL3*, *TNFSF13*, *IL-1B*, *LTA*, *OSM*, *XCL2*, *XCL1* and *PF4* (Fig. 3c). This implies various mechanisms are responsible for the inflammatory cytokine storm. Further analysis found that the top 10 most highly secreted cytokines (*S100A12, IL16, TNFSF13, XCL2, TXLNA, PF4, IL1B, CCL3, IL12RB1* and *XCL1*) contributed to >99% of the inflammatory cytokine scores in COVID-19 acute necrotizing encephalopathy patients (Fig. 3d), confirming the central role that these pro-inflammatory cytokines play to establish cytokine storm. Among the top 10 cytokines, *S100A12*, mainly secreted by the Mono_CD14 cell subset (Fig. 3d, e), may have a major role for initiating cytokine storms in COVID-19 acute necrotizing encephalopathy patients as this cytokine contributed to >96% of the cytokine score. Interestingly, significantly elevated expression of the *S100A12* gene was also seen in COVID-19 acute necrotizing encephalopathy patients (Fig. 3f and Supplementary Fig. 4f). The Mono_CD14 subtype also expressed greater cell-type-specific pro-inflammatory cytokines (such as *S100A12, CCL3, CXCL2, IL1B* and *CXCL10*), indicating a core role for this cluster in driving the inflammatory cytokine storm (Fig. 3c). Our findings highlight the importance of the Mono_CD14 subtype for designing potential therapeutic strategies to decrease immunopathogenesis in COVID-19 acute necrotizing encephalopathy patients.

*S100A12*, as a marker of inflammatory disease, is overexpressed during inflammation.^26^ This cytokine is a *TLR4* (Toll-like receptor 4) ligand and its signal transduction triggers proinflammatory activation.^26^ Relative to healthy controls and COVID-19 patients with non-acute necrotizing encephalopathy, significantly elevated expression of *TLR4* was seen in COVID-19 acute necrotizing encephalopathy patients (Fig. 3f), particularly in inflammatory monocytes (Supplementary Fig. 4g). This is consistent with previous results showing that *S100A12* activates monocytes through *TLR4* signaling.^26^ This signaling leads to the release of diverse pro-inflammatory cytokines via the *MYD88* pathway.^27^ As expected, significantly elevated expression for *MYD88* was also seen in COVID-19 acute necrotizing encephalopathy patients (Fig. 3f). These findings indicate that COVID-19 acute necrotizing encephalopathy is associated with *S100A12*-*TLR4*-induced inflammation, highlighting the importance of the pro-inflammatory *S100A12* molecule for development of potential therapeutic strategies for acute necrotizing encephalopathy. Interestingly, *S100A12*-*TLR4* signal pathway also was enriched in severe COVID-19 patients relative to mild and healthy donors (Supplementary Fig. 4j), suggesting that this signal pathway may also contribute to the development of severe disease.

We hypothesized that the inflammatory cytokine storms in COVID-19 acute necrotizing encephalopathy patients might be linked to cellular crosstalk via release of various pro-inflammatory cytokines. To evaluate this hypothesis, the patterns for different ligand-receptor pairings amongst the three hyper-inflammatory cell subsets in COVID-19 acute necrotizing encephalopathy patients were explored (Supplementary Fig. 4h). We observed notable ligand-receptor interaction between inflammatory cells from COVID-19 patients with acute necrotizing encephalopathy (Supplementary Fig. 4h). CD14-expressing monocytes (Mono_CD14) exhibited more interactions with each other compared to CD4_Memory and CD8_Pro subtypes (Fig. 3h and Supplementary Fig. 4h). Mono_CD14 cells expressed multiple receptors (e.g., *TNFRSF1A, TNFRSF1B, TNFRSF10B, DPP4, IL15RA* and *CXCR3*), suggesting that these cells can simultaneously respond to numerous cytokines secreted from other cells (Supplementary Fig. 4h). The interactions amongst Mono_CD14 and other cells appears to rely on PF4 | CXCR3, TNFSF13 | FAS, IL15 | IL2RG, TNFSF12 | TNFRSF25, NECTIN2 | CD226, CD58 | CD2, RETN | GPR25 and ALCAM | CD6 (Fig. 3h and Supplementary Fig. 4h). Together, our results demonstrate the molecular basis for potential interactions of inflammatory cells in COVID-19 acute necrotizing encephalopathy patients.

Beyond *S100A12*, we measured the circulating cytokine levels for 32 cytokines in our samples using a ProcartaPlex immunoassay. We found significant elevation of pro-inflammatory-cytokines, including IP-10, IL-6, IL-8, IL-1RA, MIP-1α/β, and MCP-1 in COVID-19 acute necrotizing encephalopathy patients (Supplementary Fig. 5). However, these pro-inflammatory cytokines were not expressed in the PBMCs from COVID-19 acute necrotizing encephalopathy patients (Fig. 3), implying that these elevated serum pro-inflammatory cytokines primarily originated at the infection site, i.e. brain or respiratory tract of patients with acute necrotizing encephalopathy.

### Dysregulated T-cell response in COVID-19 acute necrotizing encephalopathy patients

**Fig. 4:**
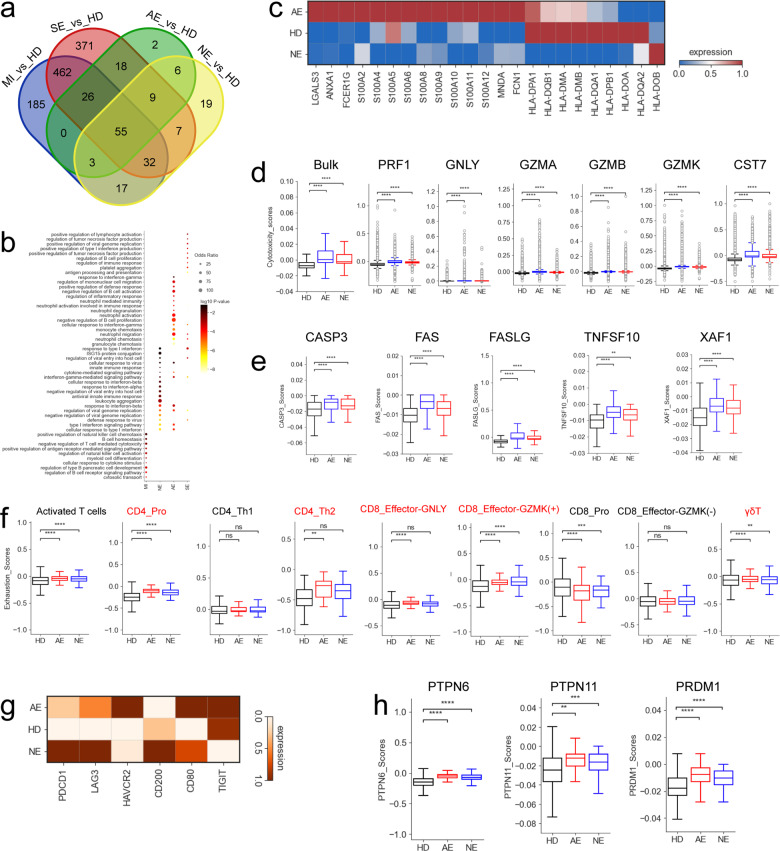
Gene expression differences in T cells from different COVID-19 groups. **a** Venn diagram shows number of upregulated DEGs in T cells, comparisons as indicated. **b** Selected enriched GO BP terms for genes upregulated in T cells. The colored bars show Log_10_ *P*-value. **c** Heatmap depicting normalized expression for selected neutrophil activation associated genes and HLA-II genes in T cells between different groups (HD, AE and NE). **d** Box plots depicting cytotoxicity scores (Left) and cytotoxicity-related genes expressed in T cells from different groups (HD, AE and NE). **e** Box plots depicting apoptosis-related gene expression in T cells between different groups (HD, AE and NE). **f** Box plots depicting exhaustion scores in effector T cells between different groups (HD, AE and NE). **g** Heatmap depicting normalized exhaustion-related gene expression in T cells between different groups (HD, AE and NE). **h** Box plots depicting selected genes expressed in T cells between different groups (HD, AE and NE). Significant differences in **d**, **e**, **f** and **h** were determined with a two-sided Student’s *T*-test with Bonferroni correction. Standard Error (SE) and median are shown

To further understand the functional status of T cells in COVID-19 patients with encephalopathy, we performed transcriptomic analysis. Among the differentially expressed genes (DEGs) in T cells, 780, 908, 119 and 148 genes were upregulated in the mild-moderate group, severe group, acute necrotizing encephalopathy and non-acute necrotizing encephalopathy groups, respectively, when compared to healthy donors (Fig. 4a and Supplementary Table 4). Of the upregulated genes, 55 genes were common to all COVID-19 patients (Fig. 4a). The GO (Geno Ontology) terms for the 55 shared upregulated genes included ‘defense response’, ‘inflammatory response’ and ‘interferon response’ (Supplementary Fig. 6a), which implies a uniform COVID-19 immune response. In addition to the antiviral responses (e.g., ‘Interferon response’, ‘Defense response to virus’ and ‘Cellular response to virus’) enriched in COVID-19 acute necrotizing encephalopathy patients, GO terms for neutrophil activation and neutrophil-mediated immune response were significantly enriched in this group compared to other COVID-19 groups (Fig. 4b). Consistently, the expression of genes involved in neutrophil activation (*S100A8*, *S100A9*, *S100A12, ANXA1* etc.) were elevated in COVID-19 patients with acute necrotizing encephalopathy (Fig. 4c). These upregulated genes and enriched GO terms in COVID-19 acute necrotizing encephalopathy patients reflect the immune response to SARS-CoV-2 infection, resulting in potential immunopathogenesis. In contrast, expression of MHC-II genes (*HLA-DQB1*, *HLA-DPB1* and *HLA-DQA1*, etc.) were decreased in COVID-19 acute necrotizing encephalopathy patients (Fig. 4c), indicating an immune paralysis of T cells. In addition, we also analyzed the DEGs between encephalopathy patients and non-encephalopathy patients, and 170 upregulated genes were shared (Supplementary Fig. 6a). Interestingly, these upregulated genes majorly involved in anti-virial response, further suggesting a uniform immune response against target virus (Supplementary Fig. 6a).

We then investigated the T cell cytotoxicity scores in different groups (Fig. 4d). T cells in COVID-19 patients with encephalopathy displayed higher cytotoxicity scores at the bulk level, with acute necrotizing encephalopathy having the highest (Fig. 4d). Five T cell subtypes: CD8_Pro, CD8_effector-GNLY, CD8_effector-GZMK^(+)^, CD8_effector-GZMK^(-)^ and γδT, had higher cytotoxic scores compared to others T cell subtypes (Supplementary Fig. 6c). COVID-19 patients with encephalopathy had significant overexpression of various cytotoxic genes, including *PRF1*, *GZMK, GZMA*, *GZMB*, *GNLY*, and *CST7* (Fig. 4d). Although the cytolytic function in T cells are required for killing virus or virus-infected cells through granule-mediated (e.g., granzyme, granulysin and perforin) functions, overexpression of these effector molecules can also result in immunopathology via inducing inflammatory response and degrading the extracellular matrix. Hence, we inferred that upregulation of different cytolytic proteins in T cells are related to the immunopathology observed in COVID-19 patients with encephalopathy, particularly in acute necrotizing encephalopathy.

Besides anti-viral responses, cytolytic molecules are also involved in apoptosis. Here, we investigated genes related to granzyme/perforin-mediated apoptosis (e.g., *GZMA*, *CASP3* and *PRF1*) (Fig. 4d). Similar to *GZMA*, *PRF1*, *GZMK* and *GZMB* in T cells, significant increases in *CASP3* were also present in COVID-19 patients with encephalopathy, and the highest expression of this gene was observed in acute necrotizing encephalopathy patients (Fig. 4e). Furthermore, significant upregulation of genes in the TNF-, FAS- and XAF1-apoptosis related pathways (e.g., *TNFSF10*, *FAS*, *FASLG* and *XAF1*) were also observed in T cells from COVID-19 encephalopathy patients, especially for those with acute necrotizing encephalopathy (Fig. 4d). Our findings indicate that upregulated FAS, TNF, XAF1 and perforin/granzyme apoptosis genes may lead to increased apoptosis of T cells, particularly in those with acute necrotizing encephalopathy. Additionally, based on apoptosis scores, we observed that CD8_Pro, CD8_eMemory, CD8_effector-GZMK^(+)^, CD8_effector-GZMK^(-)^,and γδT, T cells were more likely to have underwent apoptosis (Supplementary Fig. 6d).

Next, we examined the potential factors associated with dysfunctional T cell immune responses and immune exhaustion in COVID-19 encephalopathy. Using a collated list of exhaustion-associated markers, we defined the exhaustion score for each activated T cell. At the bulk level, there were significant elevated expression in exhaustion genes from activated T cells in the COVID-19 encephalopathy group, especially in those with acute necrotizing encephalopathy (Fig. 4f). Five T cell subtypes (CD4_Pro, CD4_Th2, CD8_Effector-GNLY, CD8_Effector-GZMK^(+)^ and γδT) were detected with higher exhaustion scores, indicating these cell subtypes may be the primary T cells under exhaustion. Further analysis identified that COVID-19 encephalopathy patients had high expression of multiple inhibitory molecules in their T cells (e.g., *PDCD1*, *LAG3* and *HAVCR2*, etc.) (Fig. 4g). PDCD1 interacts with PDL-1/PDL2 and HAVCR2 with galectin-9, which recruits *PTPN6* (also referred to as *SHP1*) and/or *PTPN11* (also referred to as SHP2) tyrosine phosphatases. This results in reduced cell proliferation and cytokine production. Interestingly, *PTPN6* and *PTPN11* expression was significantly increased in COVID-19 encephalopathy patients (Fig. 4h). Furthermore, significant increased expression of *PRDM1*, a key transcriptional factor (TF), was also found in COVID-19 encephalopathy patients relative to healthy donors (Fig. 4h). Upregulated *PRDM1* is associated with elevated expression of inhibitory receptors and decreased poly functionality in exhausted T cells. These results suggest that exhausted T cells might be driving immune dysfunction in COVID-19 encephalopathy patients, particular in acute necrotizing encephalopathy.

Similar to our observations in COVID-19 acute-encephalopathy patients, we also detected substantial expression of genes associated with neutrophil activation (Supplementary Fig. 6b), T cell apoptosis (Supplementary Fig. 6e) and exhaustion in the severe COVID-19 group (Supplementary Fig. 6f). Additionally, the elevated cell apoptosis and exhaustion observed in T-cells were also observed in NK cells from COVID-19 encephalopathy patients (Supplementary Fig. 6h).

### B cell heterogeneity in COVID-19 encephalopathy patients

**Fig. 5:**
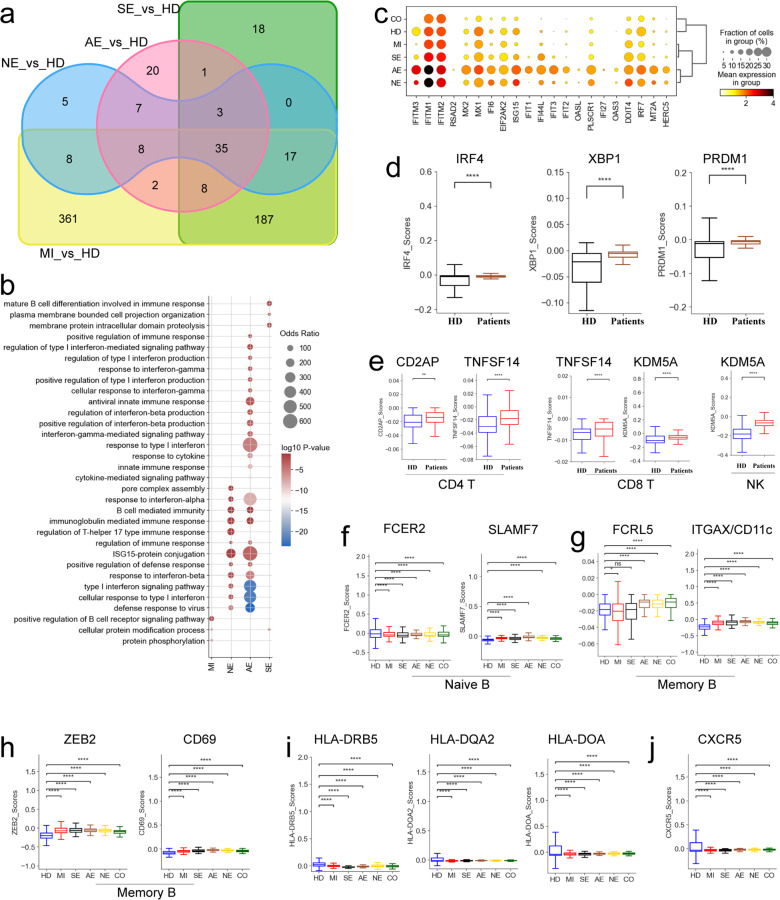
Gene expression differences in B cells from different COVID-19 groups. **a** Venn diagram shows number of upregulated DEGs in B cells, comparisons as indicated. **b** Selected enriched GO BP terms for genes upregulated in B cells. The colored bars show Log_10_ *P*-value. **c** Dot plots of selected IFN-response genes in B cells between groups. **d** Box plots of the selected genes in B cells between COVID-19 patients and healthy donors. **e** Box plots of selected genes in CD4 (Left), CD8 (Middle) and NK (Right) cells between COVID-19 patients and healthy donors. **f** Box plots depicting selected genes expressed in naïve B cells from different groups. **g** Box plots depicting selected genes expressed in memory B cells from different groups. **h** Box plots depicting *ZEB2* and *CD69* gene expression in memory B cells from different groups. **i** Box plots depicting *HLA-II* gene expression in B cells from different groups. **j** Box plots depicting CXCR5 expression in B cells from different groups. Significant differences in **d**, **e**, **f**, **g**, **h**, **i** and **j** were determined with a two-sided Student’s *T*-test with Bonferroni correction. Standard Error (SE) and median are shown

Relative to healthy donors, we found 626, 269, 84 and 83 upregulated DEGs in B cells from the COVID-19 mild-moderate, severe, acute necrotizing encephalopathy and non-acute necrotizing encephalopathy groups, respectively (Fig. 5a and Supplementary Table 5). Of the upregulated DEGs in B cells, 35 were common in all COVID-19 patients (Fig. 5a). GO analysis of these 35 shared DEGs found that terms including ‘inflammatory response’ and ‘defense response’, etc. were enriched (Supplementary Fig. 7a). Further DEG analysis showed COVID-19 associated acute necrotizing encephalopathy were characterized by enrichment of interferon (IFN) responses (e.g., ‘response to type I interferon’ and ‘response to interferon-α’, etc.) (Fig. 5b). Consistently, genes (e.g., *ISG15*, *IFITM1*/2/3, *OAS3* and *IFIT1*/2/3) associated with the IFN-response pathway, especially for IFN-I response (Supplementary Fig. 7b), were significantly upregulated in COVID-19 associated acute necrotizing encephalopathy compared with healthy donors and other COVID-19 groups (Fig. 5c). A recent report proposed that the IFN-response, especially IFN-I, exacerbates inflammation in COVID-19 patients.^28^ In addition, 79 upregulated DEGs genes were shared between encephalopathy patients and non-encephalopathy patients in B cells (Supplementary Fig. 7a), which were majorly associated with humoral immune response, further suggesting a uniform immune response in B subset (Supplementary Fig. 7a). Here, our findings suggest that the IFN-response pathway, especially IFN-I, may also contribute to the development of acute necrotizing encephalopathy in COVID-19 patients.

Next, the expression of important B-cell-activation genes in COIVD-19 were also investigated. The function and identity of plasma/cycling plasma cells are dependent upon three TFs, i.e., *IRF4*, *XBP1* and *PRDM1*. In COVID-19 patients from this study, these TFs were significantly upregulated (Fig. 5d). *IRF4* promotes the production of plasma cells and regulates Ig class-switch recombination, *XBP1* maintains protein production in plasma cells, and *PRDM1* determines the secretory arm in B cell differentiation and increases Ig production.^29^ In addition, *CD2AP* expression in CD4^+^ T cells from COVID-19 patients was significantly elevated compared with healthy donors (Fig. 5e). This gene modulates Tfh (follicular helper T) cell differentiation and during viral infection, increases the protective antibody response.^29^ Likewise, the expression of *TNFSF14*, which supports plasma cell function, on T cells (including CD4 T and CD8 T cells) was also significantly increased (Fig. 5e). Moreover, *KDM5A*, which is required for B/NK/T cell activation, was significantly increased in and CD8 T cells and NK cells (Fig. 5e). These results support that increased plasma cells (Fig. 2) and B-cell activation-related gene expression contributes to protection against COVID-19. In addition to activation of plasma-related pathways, we also observed in naïve B cells that *FCER2* was downregulated while *SLAMF7* was upregulated suggesting significant activation of these cells in COVID-19 (Fig. 5f). This is consistent with previous reports.^30^ A memory B cell population (B_Memory) which showed tissue-like memory B cell phenotype (high expression of *FCRL5* and *ITGAX*) (Supplementary Fig. 7c), was observed to be high in COVID-19 groups with severe symptoms and encephalopathy (Fig. 5g). Genes involved in activation of memory B cells, including *CD69* and *ZEB3*, higher in COVID-19 patients relative to healthy donors (Fig. 5h). These results imply that SARS-CoV-2 infection activates naïve and memory B cells. Previous studies have also observed B cell activation in COVID-19 pediatric patients relative to healthy donors.^31^

Furthermore, we found significant downregulation of most of HLA class II genes (*HLA-DRB5*, *HLA-DQB1* and *HLA-DQA2*, etc.) in B cells from COVID-19 patients (Supplementary Fig. 7d), especially in the severe and acute necrotizing encephalopathy groups (Fig. 5i), indicating dysregulated crosstalk between adaptive immune cells. Furthermore, we found that the chemokine receptor *CXCR5* was significantly decreased in COVID-19 patients, which might lead to inhibition of germinal center reactions and the dysregulated humoral immune responses observed in the early stage of SARS-CoV-2 infection.^30^

### Remodeling of circulating monocytes in COVID-19 encephalopathy patients

**Fig. 6:**
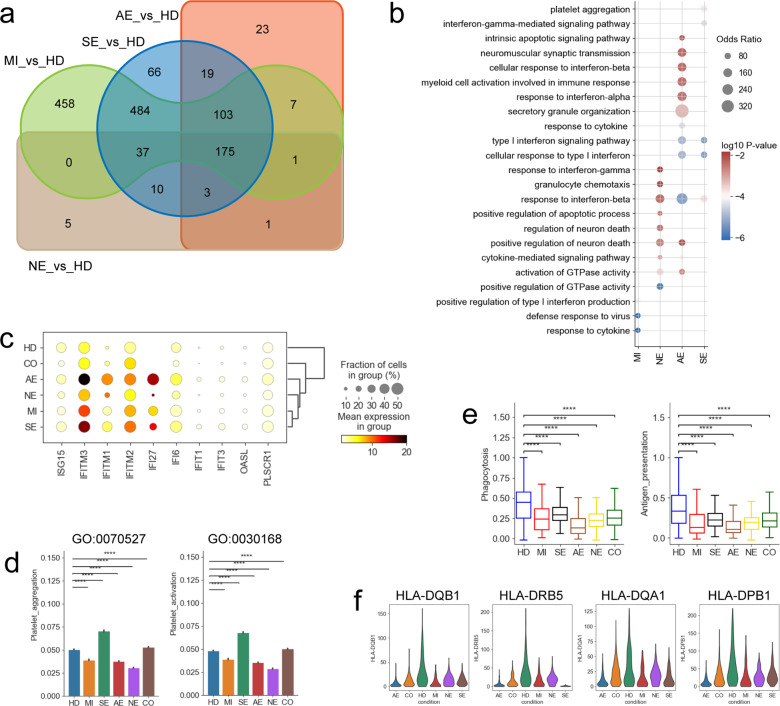
Gene expression differences in myeloid cells from different COVID-19 groups. **a** Venn diagram shows number of upregulated DEGs in myeloid cells, comparisons as indicated. **b** Selected enriched GO BP terms for genes upregulated in myeloid cells. The colored bars show Log_10_ *P*-value. **c** Dot plots of selected IFN-response genes in myeloid cells between groups. **d** Bar plots of the normalized expression score for two GO gene sets (platelet_aggregation (GO:0070527) and platelet_activation (GO:0042113)) in megakaryocytes. **e** Box plots of phagocytosis scores and antigen presentation scores in DCs. **f** Violin plots of representative HLA-II genes in DCs from different COVID-19 groups. Significant differences in **d**, **e** were determined with a two-sided Student’s *T*-test with Bonferroni correction. Standard Error (SE) and median are shown

Monocytes are one of the primary peripheral myeloid cells. Four monocyte subtypes were identified in this study (Mono_CD14, MDSCs, Mono_C1QA and Mono_CD14_CD16) (Fig. 2a). We first focused on classical monocytes (Mono_CD14) due to their central role in inflammatory responses (Fig. 3). Mono_CD14 cells were significantly elevated in the COVID-19 severe disease and acute necrotizing encephalopathy groups (Supplementary Fig. 8a). Among the DEGs in CD14^+^ monocytes, 1265, 899, 332 and 232 genes were upregulated in COVID-19 patients with mild-moderate disease, severe disease, acute necrotizing encephalopathy and non-acute necrotizing encephalopathy, respectively, of which, 175 were shared (Fig. 6a and Supplementary Table 6). Similar to our observations in B cells, GO terms associated with the IFN response (e.g., type I interferon signaling pathway, response to interferon-α, etc.), especially IFN-I response, were enriched in CD14^+^ monocytes in COVID-19 acute necrotizing encephalopathy patients relative to healthy controls and other COVID-19 groups (Fig. 6b). When analyzed further, COVID-19 acute necrotizing encephalopathy patients also had various ISGs upregulated (e.g., *ISG15*, *IF27* and *IFITM1*/*2*/*3* etc.), consistent with our GO analysis (Fig. 6c). Further analysis found that monocytes, especially classical CD14^+^ monocytes (Mono_CD14), were the most prominent cell subtype associated with the type I response (Supplementary Fig. 8b, c). These data indicate that in addition to *S100A12*-*TLR4*-inflamamtory features, COVID-19 patients with acute necrotizing encephalopathy also acquires *IFN-I*-responsive features (Figs. 3, 6). We inferred that the *IFN* response may potentiate *S100A12*-*TLR4*-driven inflammation thus contributing to the hyper-inflammatory response. Similar findings showing that *IFN-I* strengthens *TNF*- and *IL-1β*-induced inflammation in COVID-19 severe disease have been reported.^28^ Consistent with our scRNA-seq data, we found a significant increase in IFN-α plasma concentration in COVID-19 patients with acute necrotizing encephalopathy (Supplementary Fig. 5). Interestingly, GO terms, including positive regulation of neuron death and regulation of neuron death were also more enriched in COVID-19 patients with encephalopathy (Fig. 6b), indicating that classical monocytes might have a direct role in the development of COVID-19-associated encephalopathy.

Megakaryocytes are involved in hemostasis, however it is unknown if they contribute to COVID-19-associated encephalopathy. Two GO pathways (GO: 0070527 and GO:0030168) associated with platelet aggregation and activation were significantly upregulated in severe COVID-19 disease (Fig. 6d). In contrast, we did not observe upregulated platelet aggregation/activation in COVID-19 encephalopathy patients (Fig. 6d), indicating a low risk of thrombosis in these patients. Megakaryocytes can also contribute to inflammatory signaling however no upregulation of inflammatory-related genes (e.g., *CCL2*, *CLL3*, *IL-6* and *CXCL8*, etc.) were observed in the megakaryocytes from COVID-19 patients including those with encephalopathy (Supplementary Fig. 8d). This was consistent with our earlier results (Fig. 3, Supplementary Fig. 4) that this subtype was not a peripheral source of pro-inflammatory cytokines and suggests that megakaryocytes are not major contributors in the pathogenesis of COVID-19-associated encephalopathy.

DCs are important antigen presenting cells which activate the adaptive immune response including T cells. Here, the DC phagocytosis and antigen presenting capacity in COVID-19 disease were examined. We observed that DCs from COVID-19 patients showed reduced phagocytosis capacity relative to healthy donors (Fig. 6e). There was also reduced expression of phagocytosis-related genes (e.g., *CDC42*, *WASF2*, *RAC2* and *VASP*, etc.) (Supplementary Fig. 8e). Consistently, DCs from COVID-19 patients also displayed lower antigen presentation capacity than healthy donors (Fig. 6e), with genes associated with antigen presentation (e.g., *HLA-DQB1*, *HLA-DEB5* and *HLA-DQA1*, etc.) downregulated in DCs from the COVID-19 groups including those with encephalopathy (Fig. 6f and Supplementary Fig. 8f). These results suggest that DCs contribute to immune paralysis in COVID-19 patients including in those displaying encephalopathy.

## Discussion

COVID-19 remains a significant public health threat. A range of clinical manifestations from asymptomatic, mild or moderate to severe disease can be caused by SARS-CoV-2 infection. Recently, many studies have reported COVID-19-associated severe neurological complications including encephalopathy (e.g., acute necrotizing encephalopathy).^7^ COVID-19 patients with encephalopathy, especially those with acute necrotizing encephalopathy, require ICU (Intensive Care Unit) admission and are associated with high mortality rates. Currently, there is still a lack of comprehensive understanding on the immune response in COVID-19-associated encephalopathy. This knowledge is fundamental for designing effective treatment strategies, predicting disease prognosis, and achieving further cognition of heterogeneity of COVID-19-associated encephalopathies. Here, we utilized scRNA-seq to integrate clinical observations (Supplementary Table 1) and laboratory examinations (Supplementary Fig. 5), to develop an integrated and comprehensive understanding of COVID-19 encephalopathy, including acute necrotizing encephalopathy and non-acute necrotizing encephalopathy.

A rich scRNA-seq dataset was produced from 22 PBMC samples collected from 17 pediatric patients, providing a valuable source of information to dissect the immune response in COVID-19 encephalopathy at the single-cell level and highlight important immune features of this disease. Eight major cell clusters and 30 cell subtypes were identified (Figs. 1, 2), providing detailed insights into the molecular and cellular responses in COVID-19 encephalopathy patients. Overall, COVID-19 associated encephalopathy, especially for acute necrotizing encephalopathy, had a strong effect on the composition of peripheral immune cells. We identified a sharp reduction in the number of lymphocytes in acute necrotizing encephalopathy patients (Figs. 1, 2 and Supplementary Fig. 1–4). Our results suggest that lymphopenia may be a typical feature of COVID-19-associated acute necrotizing encephalopathy. Multiple lymphocytes populations, such as CD8^+^T, CD4^+^T, γδT and NK cells, were affected by the lymphopenia phenomenon with T-cells preferentially impacted in acute necrotizing encephalopathy (Figs. 1, 2 and Supplementary Fig. 1–3). T cells and its subtypes were significantly reduced in COVID-19 acute necrotizing encephalopathy patients and exhibited a notable association with this disease (Figs. 1, 2). It remains unclear why lymphopenia in COVID-19 acute necrotizing encephalopathy patients is biased towards T cells and the underlying mechanisms of lymphopenia requires further study.

Lymphopenia has been reported in various respiratory viral infections (e.g., SARS-CoV-2, human rhinovirus, influenza virus, and RSV, etc.), particularly in patients with severe disease.^18^ However, the mechanisms of lymphopenia remain incompletely understood in these infectious diseases, including in COVID-19. Previous studies have suggested that lymphopenia might be related to high levels of IL-10 or IL-6, potentially through a direct role of these cytokines on lymphocytes and/or indirect roles via other cell lineages (e.g., neutrophils and DCs).^32–35^ Interestingly, we observed elevated levels of IL-6 and IL-10 in the COVID-19 AE group (Supplementary Fig. 5). It is also possible that the peripheral lymphopenia seen in the COVID-19 AE group may reflect recruitment of lymphocytes (e.g., B, NK, T cells) to the infection sites. Unfortunately, we did not collect the cerebrospinal fluid and lung tissue from those patients, it thus remains elusive whether lymphopenia is also caused by tissue infiltration. In addition, the activation of various apoptosis-associated pathways (e.g., FAS-induced apoptosis pathway) could also contribute to lymphocyte depletion.^25^ Consistently, various pro-apoptotic molecules (e.g., *CASP3*, *FAS*, *FASLG* and *XAF1*, etc.) were highly expressed in the COVID-19 AE group, implying that cell apoptosis might be related to lymphopenia observed in patients with AE.

In contrast to lymphocytes, various myeloid cells (including megakaryocytes and monocytes) were increased, suggesting a severe inflammatory response (Figs. 1, 2). Recent reports suggested that inflammation is an important cause for COVID-19 associated encephalopathy.^7^ We demonstrated the presence of cytokine storms in COVID-19 acute necrotizing encephalopathy patients (Fig. 3), which might be related to disease severity and immunopathogenesis. We identified the cellular source for pro-inflammatory cytokine storm in COVID-19 acute necrotizing encephalopathy patients as primarily originating from three cell subtypes, a CD14^+^ monocyte subset (Mono_CD14), a proliferative CD8 T subset (CD8_Pro) and a CD4 memory T subset (CD4_Memory) (Fig. 3, Supplementary Fig. 4). Although many inflammatory cytokines were elevated in acute necrotizing encephalopathy patients, *S100A12*, mainly secreted by Mono_CD14 cells (Fig. 3), may be a key player for driving the inflammatory cytokine storm. Similar with a previous report,^31^ classical monocytes displayed significantly higher levels of S100A family inflammatory genes (e.g., *S100A12*) in COVID-19 pediatric patients with relatively severe symptoms. Various inflammatory cells (e.g., granulocytes and monocytes) overexpress *S100A12* and the elevated serum levels of this molecule has been found in patients with different inflammatory diseases, including viral infections.^26^ In support of this, we confirmed that *S100A12* was significantly overexpressed in COVID-19 patients with acute necrotizing encephalopathy (Fig. 3). Extracellular *S100A12* protein is known to cause pro-inflammatory responses through *TLR4*. As expected, *TLR4* was significantly upregulated in COVID-19 acute necrotizing encephalopathy patients, mainly in inflammatory monocytes (Supplementary Fig. 4). This could further promote *S100A12*-mediated inflammation. The blocking of *S100A12* from binding to *TLR4* may inhibit the downstream pro-inflammatory signal and is therefore of potential value for designing effective therapeutics against COVID-19-associated acute necrotizing encephalopathy. The use of anti-*S100A12* treatments could be beneficial to patients with acute necrotizing encephalopathy because modulating production of this molecule might blunt the ‘cytokine storm’. In addition, the upregulation of *S100A12*-*TLR4* signal pathway also were observed in severe patients, indicating that *S100A12*-*TLR4*-inflammatory response also was one of the important factors for driving the development of severe disease, because those patients with AE required ICU admission and also were defined as severe disease.

In addition to the *S100A12*-*TLR4*-inflammatory response, COVID-19 acute necrotizing encephalopathy patients were accompanied with an *IFN-1* response, dominant in classical monocytes (Mono_CD14). The *IFN-1* response was not present in those with non-acute necrotizing encephalopathy. Our study investigated *IFN-I* driven inflammation and found that the *IFN-I* responsive genes (e.g., *ISG15* and *IFITM1/2/3*, etc.) and associated-GO pathways were enriched in COVID-19 patients with acute necrotizing encephalopathy. Although *IFN-I* plays a role in antiviral activity, its immunopathological role has been observed previously.^36^ It was reported that a delayed and dysregulated *IFN-I* response was responsible for pathological inflammation and increased the risk of lethality in coronavirus infections, including for SARS-COV-2.^28,37,38^ A recent report validated that a dysregulated *IFN-I* response coupled with pro-inflammatory cytokine responses (e.g., *TNF* response) can potentiate the hyperinflammatory response in progression to severe COVID-19 disease.^28^ On the basis of our results and previous findings, we inferred that the *IFN-I* response might play a key role in exacerbating COVID-19 inflammatory storm, further contributing to the development of COVID-19 associated acute necrotizing encephalopathy. Therefore, inhibiting the *IFN-I* response may be crucial in improving the outcomes for COVID-19 acute necrotizing encephalopathy patients.

Monocytic MDSCs, a subtype of myeloid cells characterized by reduced *MHC-II* expression and increased neutrophil activation-associated genes (e.g., *S100A8/A9*), was identified to be significantly elevated in COVID-19 patients with encephalopathy, especially acute necrotizing encephalopathy (Fig. 2 and Supplementary Fig. 3). MDSCs are known to expand in a diverse range of inflammatory conditions.^39^ As a population of heterogeneous immature monocytes, MDSCs are important in suppressing T cells by expressing high levels of inhibitory receptor (e.g., PDL-1).^24^ Hence, we propose that for COVID-19, these cells dampen the host immune response and potentiate COVID-19-associated encephalopathy pathogenesis. Interestingly, similar data and conclusions have been recently reported by several independent studies in severe COVID-19 patients,^40,41^ which further corroborates our hypothesis. Besides increased MDSCs, we also observed that DCs were depleted and their phagocytosis and antigen presentation capacity downregulated, further suggesting peripheral immune paralysis in COVID-19 encephalopathy patients. Interestingly, pDCs were reduced in patients with severe disease and NE (Fig. 2); similar observations were seen for COVID-19 pediatric patients with multisystem inflammatory syndrome.^31^ Collectively, our scRNA-seq analysis suggests that myeloid cells are involved in suppressing host immune responses in COVID-19 encephalopathy patients.

Additionally, dysregulated T, NK and B cell responses were observed in COVID-19 acute necrotizing encephalopathy patients, which may also explain why these patients fail to control COVID-19. We showed that firstly, T and NK cells were exhausted in COVID-19 acute necrotizing encephalopathy patients, and this was evidenced by (i) increased expression of multiple inhibitory receptors and (ii) elevated expression of exhaustion-related transcription factors. T/NK cell exhaustion is often related to ineffective control of pathogen infections and tumors, thus modulating pathways overexpressed in exhaustion (e.g., by targeting *PD1*) may be able to reverse the dysfunctional state and restore effective immune responses. Secondly, various cytotoxic molecules (e.g., *PRF1*, *GZMA* and *GNLY*, etc.) that were highly expressed in T and NK cells might also be associated with the immunopathology in COVID-19 acute necrotizing encephalopathy patients, because these effector molecules can damage multiple organs, including the CNS, by eliciting an inflammatory response and degrading the extracellular matrix. Thirdly, genes involving in neutrophil-mediated immunopathology (e.g., *S100A8*, *S100A9* and *S100A12*, etc.) and pro-inflammatory responses were elevated in COVID-19 acute necrotizing encephalopathy patients. This may reflect the host immune responses to SARS-CoV-2 and result in immunopathogenesis. Fourthly, increased apoptosis of T and NK cells were seen in COVID-19 acute necrotizing encephalopathy patients and may be an explanation for the lymphopenia observed in these patients. Fifthly, for B cells, significant downregulation of *MCH-II* molecules and loss of chemokine receptor *CXCR5* were seen in COVID-19 patients with acute necrotizing encephalopathy. This may cause dysregulation of humoral immune responses including impairing crosstalk between the adaptive immune cells and germinal center responses. Taken together, our results demonstrate a dysregulated T, NK and B cell response in COVID-19 acute necrotizing encephalopathy patients.

Age-associated qualitative and quantitative changes in the immune system might affect immune cells and soluble mediators of both the adaptive and innate immune responses. These changes may determine disease progression of COVID-19, including pediatric patients, and clinical outcomes thereafter. In our study, pediatric patients across a broad age spectrum (1–10 years) (Supplementary Table 1) were included, such variability thus may influence the identified transcriptional profiles in this study.

In conclusion, our scRNA-seq study, which included COVID-19 pediatric patients with a range of disease severity and encephalopathy complications, revealed multiple immune hallmarks for COVID-19-associated encephalopathy that were previously unknown. Importantly, our study highlights the pathogenic features and host immune responses in COVID-19 encephalopathy patients (especially for those with acute necrotizing encephalopathy) that are suitable targets for therapy.

## Methods

### Ethical approval

The ethics for this study was approved by the Ethics Committee of the Sanya People’s Hospital (SYPH‐2021‐26) and Capital Institute of Pediatric (SER-2023-19 and SHERLL2023009).

### Study design and participants

Six healthy pediatric donors, including 3 males and 3 females, were recruited from the Sanya People’s Hospital in Aug 2021. The 6 healthy donors had no prior history of SARS-CoV-2 infection and did not receive any COVID-19 vaccines. Eleven pediatric patients diagnosed with COVID-19 were enrolled from the Capital Institute of Pediatrics. This included 2 patients displaying mild/moderate symptoms, 2 patients displaying severe symptoms but no signs of encephalopathy, 4 patients diagnosed with acute necrotizing encephalopathy and 3 patients diagnosed with non-acute necrotizing encephalopathy. Pediatric patients were classified into mild and severe groups based on the Tenth Revised Trial Version of the Novel Coronavirus Pneumonia Diagnosis and Treatment Guidelines while diagnosis of acute necrotizing encephalopathy was confirmed according to the Japanese Society of Child Neurology guidelines^42^ and a recent review.^7^ In particular, clinical features, chest radiographs and laboratory findings, which were used for defining the disease severity and encephalopathy, are listed in Supplementary Table 1. All clinical data were collected from Capital Institute of Pediatrics electronic medical records.

### Single-cell RNA sequencing and data analysis

Peripheral blood mononuclear cells (PBMCs) were isolated from fresh blood samples (*n* = 22) using standard density gradient centrifugation^16^ and cell viability >90% confirmed with the Countstar cell viability kit. 5’ libraries for single-cell RNA sequencing were prepared using the Chromium Single Cell 5’ Kit v2 (10x Genomics; PN-1000263) according to the instructions from the manufacturer. Single-cell RNA sequencing was performed Illumina Novaseq 6000 sequencer (2x150bp).

The resulting scRNA-seq data was analyzed as described previously.^1,9,16^ In brief, the merged filtered gene expression matrix for the 22 samples was obtained using kallisto/bustools (kb v0.24.4) and the ad.concat function^29^ in anndata (ad) (v0.7.6). Scanpy (sc) (v1.9.2) was then used to remove doublets/low quality cells, normalize library size to 10,000 reads per cell and select a consensus set of the top 1,500 most highly-variable genes (HVGs) with high cell to cell variation.^9,29^ Dataset integration was performed using principal component analysis (PCA) to reduce the dimensions down to 20 PCA components, Harmony algorithm implementation for batch effect correction,^43^ and Harmony and unsupervised clustering of single cell data using Louvain algorithm.^44,45^ PCA of sample variables was performed and visualized with the R package factoextra.

### Cell clustering and annotations

Two rounds of unsupervised cell clustering using the neighborhood relations of cells were performed with the sc.tl.louvain function. Eight major cell types (B cells, CD8^+^ T cells, CD4^+^ T cells, NK, gamma delta T, megakaryocytes, monocytes and dendritic cells) were identified in the first round (Louvain resolution = 2.0). The B, NK, CD4 + /CD8 + T, monocyte and DC cell types were then further sub-divided into sub-clusters in the second round (Louvain resolution 2.0). These sub-clusters represent distinct immune cell lineages inside the major cell types and was manually confirmed with canonical marker genes (Supplementary Table 2). The sc.tl.rank_genes_groups function was used to identify cluster-specific signature genes (Supplementary Table 2) which were then manually matched to canonical marker genes for cluster annotation.

### Identifying changes in immune cell proportion

The proportion for each immune cell type/subtype in different disease conditions was calculated and statistical significance confirmed using Student’s *T*-test. We also performed multivariate ANOVA to determine how different disease conditions and their potential interactions impact the proportion of each cell type/subtype.^25^ We then further calculated the disease preference for each type/subtype using R_O/E_ (the ratio of observed vs randomly expected cell numbers).^25^

### Determining cell state scores

Pre-defined gene sets were used to compare the overall activation level or physiological activity of different cell types/subtypes. The pro-inflammatory cytokine and inflammatory response gene sets were collected from published reports^16,29^ and MsigDB database for response to type I interferon (GO:0034340), response to interferon beta (GO:0035456), response to interferon alpha (GO:0035455), cellular response to type I interferon (GO:0071357), platelet activation (GO:0030168) and platelet aggregation (GO:0070527). The cytotoxicity and exhaustion scores were defined using 17 and 11 genes, respectively.^25,46^ The cell state score was defined as the average gene expression of the predefined gene set divided by the reference genes and was calculated using the sc.tl.score_genes function. A student’s *t*-test was used to test the statistical significance of cell state scores between disease conditions.

### Plasma cytokine assays

The Th1/Th2 34-plex human ProcartaPlex kit (Thermo Fisher Scientific) was used to measure plasma cytokine levels and performed as described by the manufacturer’s instructions and previous reports.^29,47^

### Statistical analysis

Python and R were used to perform all statistical analysis and visualizations. For all Figures, the following symbols are used to denote statistical significance: ns: *p* > 0.05; *p ≤ 0.05; **p ≤ 0.01; ***p ≤ 0.001; ****p ≤ 0.0001.

### Supplementary information

Supplemental main text

Supplementary Table 1

Supplementary Table 2

Supplementary Table 3

Supplementary Table 4

Supplementary Table 5

Supplementary Table 6

## Data Availability

Correspondence and requests for the data should be addressed to Y.W. (Capital Institute of Pediatrics).

## Materials availability

Correspondence and requests for the materials should be addressed to Y.W. (Capital Institute of Pediatrics).

## Code availability

Customized scripts used for analyzing data in this study are provided upon reasonable request.
