# Supplementary material for: Single-cell transcriptomic analysis reveals a systemic immune dysregulation in COVID-19-associated pediatric encephalopathy
Source: Signal Transduct Target Ther. 2023 Oct 18;8:398. doi: 10.1038/s41392-023-01641-y (PMC10582072; doi:10.1038/s41392-023-01641-y)
Supplement: Supplementary file 1 — Supplemental main text [file 41392_2023_1641_MOESM1_ESM.pdf]

Supplementary Materials for

Single-cell transcriptomic analysis reveals a systemic immune dysregulation in COVID-19-associated pediatric encephalopathy

Yi Wang [1, *, ✉], Laurence Don Wai Luu [2, *], Shuang Liu [3,*], Xiong Zhu [4, *], Siyuan Huang [3,*], Fang Li [3], Xiaolan Huang [1], Linying Guo [3], Jin Zhang [3], Haiyan Ge [3], Yuanyuan Sun [3], Yi Hui [3], Yanning Qu [3], Huicong Wang [3], Xiaoxia Wang [4], Weilan Na [3], Juan Zhou [1], Dong Qu [3, ✉] and Jun Tai [5, ✉]

Correspondence to: wildwolf0101@163.com; qudong2012@126.com; trenttj@163.com;

The file includes:

Supplementary Figure 1-8 with their legends

a

		1	2	3	4	5	6	7	8	9	10	11	12	13	14	15	
Acute necrotizing encephalopathy	P1		AE1							AE1_1						CO1	Survial
	P2		AE2														Death
	P3	AE3															Death
	P4				AE4												Death
Non-acute necrotizing encephalopathy	P5			NE1												CO2	Survial
	P6		NE2													CO3	Survial
	P7				NE3											CO4	Survial
Mild	P8				MI1												Survial
	P9				MI2												Survial
Severe	P10			SE1													Survial
	P11		SE2														Survial
Sampling days after illness onset		1	2	3	4	5	6	7	8	9	10	11	12	13	14	15	Outcomes

Note*: COVID-19 patients with encephalopathy or with severe symptoms were collected from ICU.

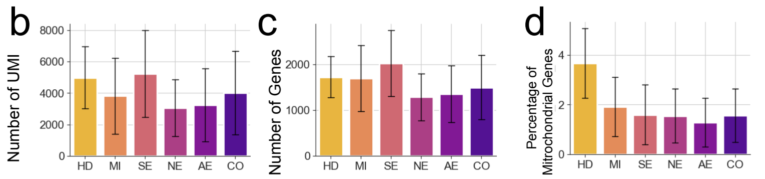

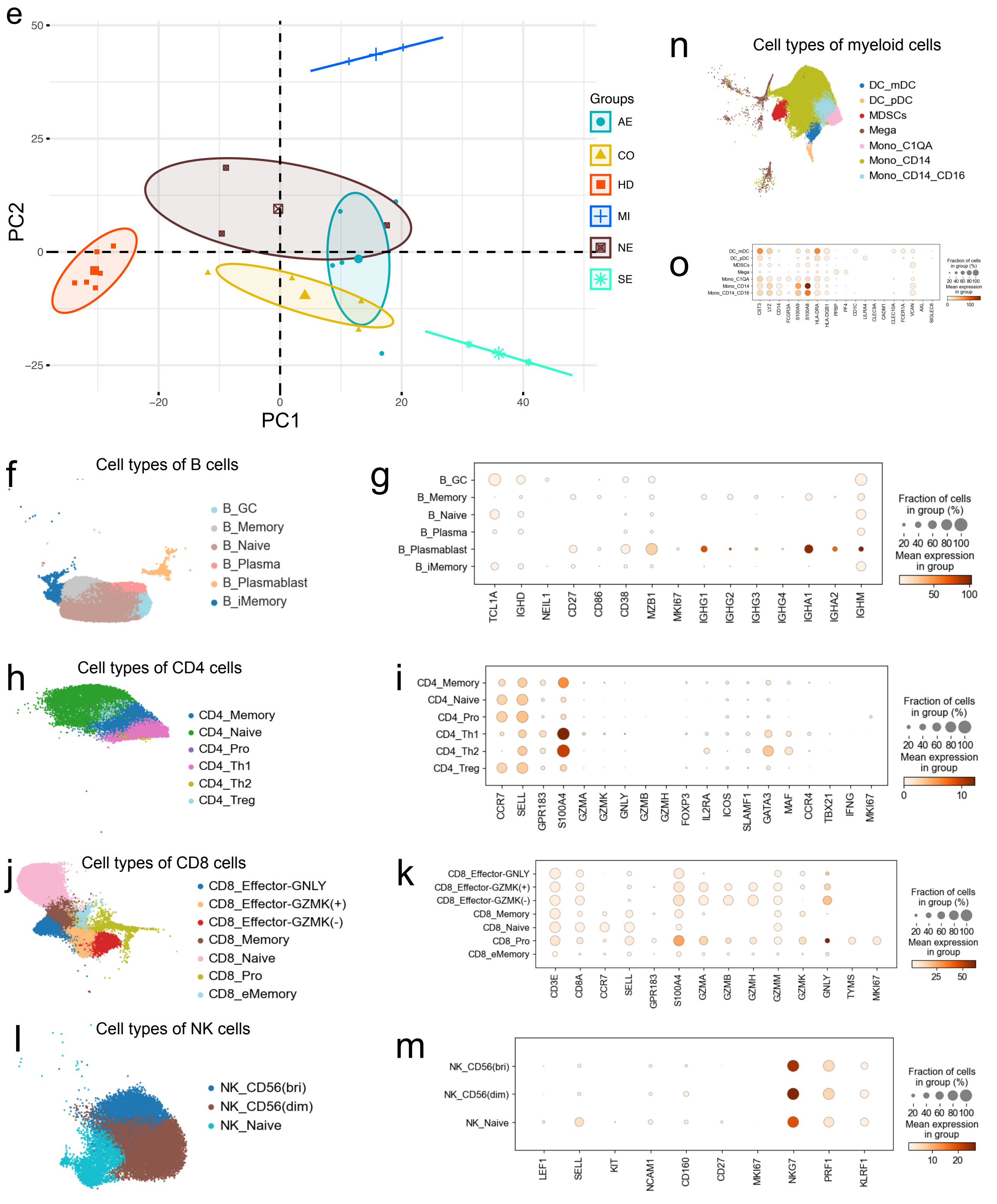

Supplementary Fig 1. Characteristics of the integrated dataset and selected markers for cell sets/subsets in different cell lineages, related to Figure 1.

a. Timeline of blood sample collection in the 11 subjects.

b-d. Distribution of the unique molecular identifier (UMI) counts per cell (b), gene counts per cell (d), and percentage of mitochondrial transcripts per cell (d) detected for cells in each disease group. The error bars represent Standard Error (SE).

e. A visualization of principal component analysis (PCA) of sample variables. Each point represents one sample (green circle for AE, yellow triangle for CO, red square for HD, blue cross for MI, brow rectangle for NE, light blue asterisks for SE). The slightly larger shape represents the group means. The ellipses around the group mean represent the confidence regions.

f, h, j, l, n. The clustering result of B (f), $CD4^{+}T$ (h), $CD8^{+}T$ (j), NK (l) and Myeloid (n) cell subsets. Each point represents one single cell, colored according to cell type.

g, i, k, m, o. Dot plots of selected marker genes (Rows) for cell subsets (Columns) within each cell lineage, including B (g), $CD4^{+}T$ (i), $CD8^{+}T$ (k), NK (m) and Myeloid (o) cell subsets.

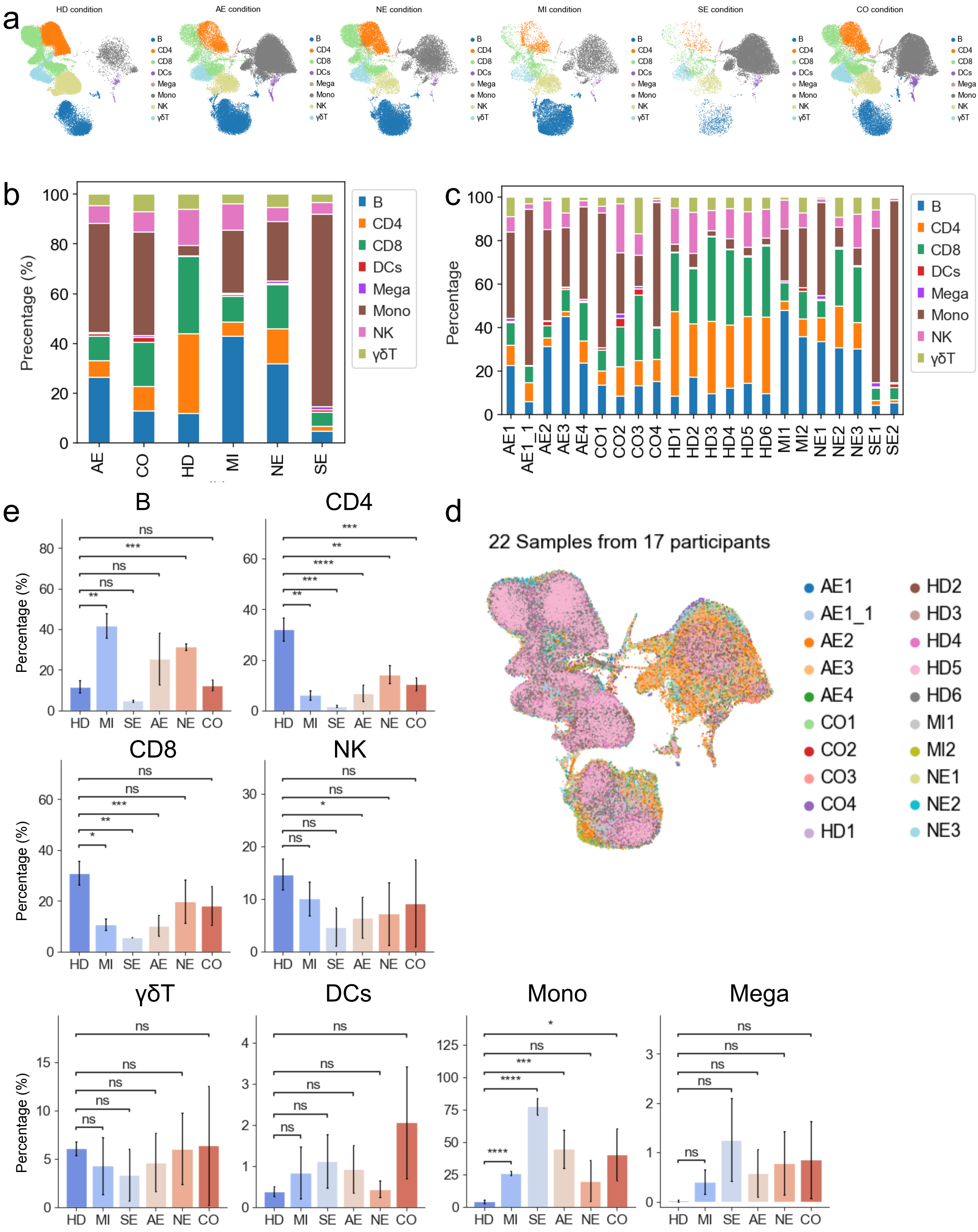
a
HD condition
AE condition
NE condition
MI condition
SE condition
CO condition
B
CD4
CD8
DCs
Mega
Mono
NK
γδT
b
Precentage (%)
AE
CO
HD
MI
NE
SE
c
Percentage
AE1
AE1_1
AE2
AE3
AE4
CO1
CO2
CO3
CO4
HD1
HD2
HD3
HD4
HD5
HD6
MI1
MI2
NE1
NE2
NE3
SE1
SE2
d
22 Samples from 17 participants
e
B
CD4
CD8
NK
γδT
DCs
Mono
Mega
Percentage (%)
HD MI SE AE NE CO
ns
*
**
***
****

Supplementary Fig 2. Detailed data output and visualization of single-cell transcriptomic profiling of PBMCs from 22 samples, related to Figure 1.

a. The UMAP projection for the six conditions on different panels. Cells are colored by the 8 major cell types.

b. Stacked bar plot showing the relative proportion of the 8 cell subtypes derived from HD, MI, SE, NE, AE and CO conditions.

c. Stack bar plot showing the relative proportion of the 8 cell subtypes for each of the 22 samples.

d. The UMAP projection for the 22 individual samples with different colors. Cells are colored according to each sample.

e. The distribution of each immune cell type across 6 conditions. The y-axis shows the average percentage of each immune cell type. Conditions are displayed in different colors on the *x* axis.

Significant differences in d, e, f and h were determined by Two-sided Student's T-test with Bonferroni correction (*p<0.05, **p<0.01, ***p<0.001, ****p<0.0001, ^{ns}p>0.05). The error bars represent Standard Error (SE), and the median is shown as horizontal bars.

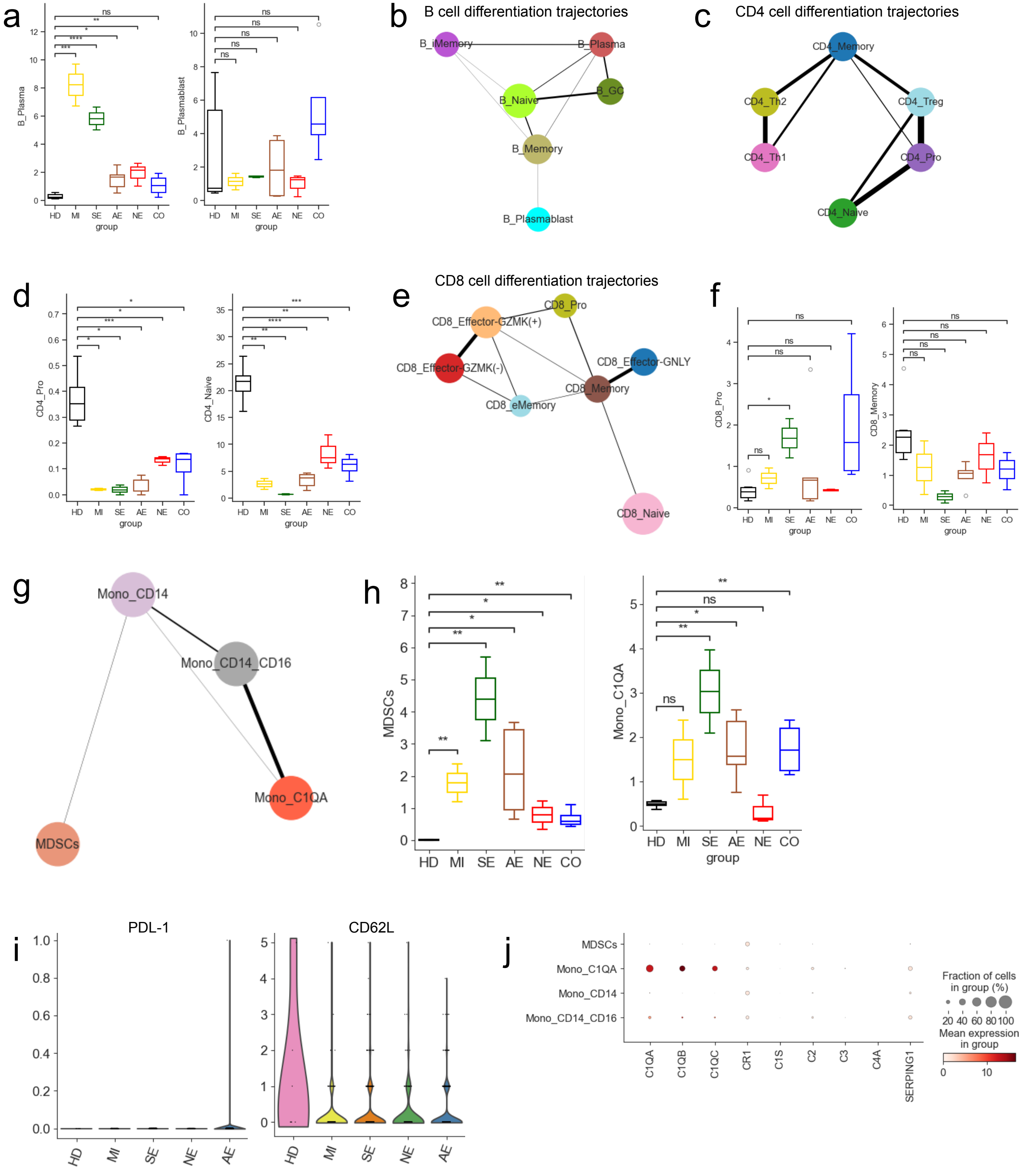

a
B_Plasma
B_Plasmablast
group
HD MI SE AE NE CO
ns
**
*
****
***
b
B cell differentiation trajectories
B_iMemory
B_Plasma
B_Naive
B_GC
B_Memory
B_Plasmablast
c
CD4 cell differentiation trajectories
CD4_Memory
CD4_Th2
CD4_Treg
CD4_Th1
CD4_Pro
CD4_Naive
d
CD4_Pro
CD4_Naive
e
CD8 cell differentiation trajectories
CD8_Pro
CD8_Effector-GZMK(+)
CD8_Effector-GZMK(-)
CD8_Effector-GNLY
CD8_Memory
CD8_eMemory
CD8_Naive
f
CD8_Pro
CD8_Memory
g
Mono_CD14
Mono_CD14_CD16
Mono_C1QA
MDSCs
h
MDSCs
Mono_C1QA
i
PDL-1
CD62L
HD MI SE NE AE
j
MDSCs
Mono_C1QA
Mono_CD14
Mono_CD14_CD16
C1QA C1QB C1QC CR1 C1S C2 C3 C4A SERPING1
Fraction of cells in group (%)
20 40 60 80 100
Mean expression in group
0 10

Supplementary Fig 3. Comparison of different immune cell types among patient groups, related to Figure 2.

a. The distribution of B_Plasma and B_Plasmablast across 6 conditions.

b. PAGA analysis of B cell pseudo-time: the associated cell type and the corresponding status are listed.

c. PAGA analysis of $CD4^+$ T cell pseudo-time: the associated cell type and the corresponding status are listed.

d. The distribution of CD4_Pro and CD4_Naive across 6 conditions.

e. PAGA analysis of $CD8^+$ T cell pseudo-time: the associated cell type and the corresponding status are listed.

f. The distribution of CD8_Pro and CD4_Memory across 6 conditions.

g. PAGA analysis of monocyte cell pseudo-time: the associated cell type and the corresponding status are listed.

h. The distribution of MDSCs and Mono_C1QA across 6 conditions.

i. Violin plots of selected genes for MDSCs across different conditions.

j. Dot plots of selected marker genes (Rows) for monocyte subsets (Columns) within each cell lineage,

Significant differences in a, d, f and h were determined by Two-sided Student's T-test with Bonferroni correction (*p<0.05, **p<0.01, ***p<0.001, ****p<0.0001, ^{ns}p>0.05). The error bars represent Standard Error (SE), and the median is shown as horizontal bars.

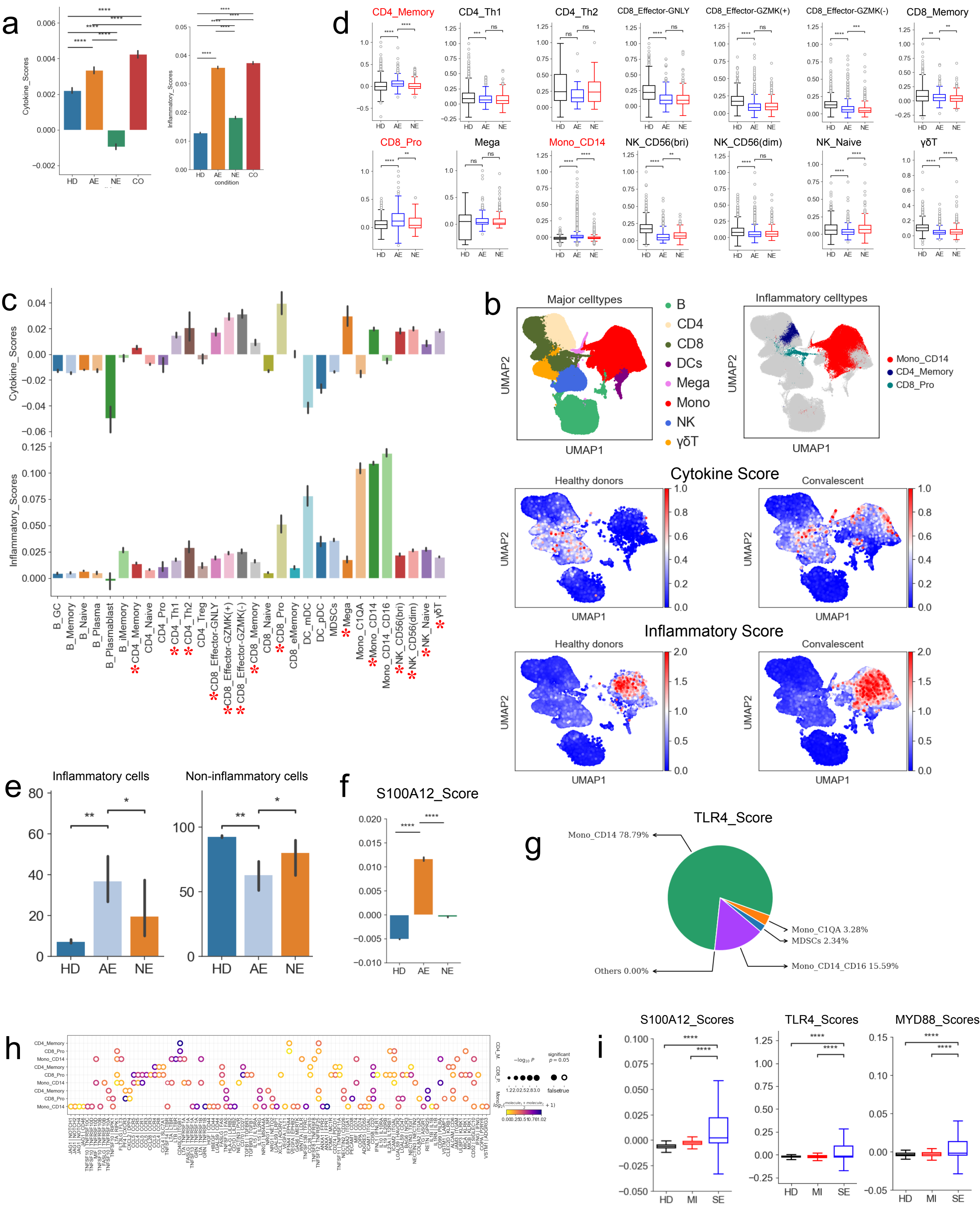

a
Cytokine_Scores
Inflammatory_Scores
HD AE NE CO
condition
d
CD4_Memory
CD4_Th1
CD4_Th2
CD8_Effector-GNLY
CD8_Effector-GZMK(+)
CD8_Effector-GZMK(-)
CD8_Memory
CD8_Pro
Mega
Mono_CD14
NK_CD56(bri)
NK_CD56(dim)
NK_Naive
γδT
HD AE NE
c
Cytokine_Scores
Inflammatory_Scores
B_GC
B_Memory
B_Naive
B_Plasma
B_Plasmablast
B_iMemory
CD4_Memory
CD4_Naive
CD4_Pro
CD4_Th1
CD4_Th2
CD4_Treg
CD8_Effector-GNLY
CD8_Effector-GZMK(+)
CD8_Effector-GZMK(-)
CD8_Memory
CD8_Naive
CD8_Pro
CD8_eMemory
DC_mDC
DC_pDC
MDSCs
Mega
Mono_C1QA
Mono_CD14
Mono_CD14_CD16
NK_CD56(bri)
NK_CD56(dim)
NK_Naive
γδT
b
Major celltypes
B
CD4
CD8
DCs
Mega
Mono
NK
γδT
Inflammatory celltypes
Mono_CD14
CD4_Memory
CD8_Pro
UMAP1
UMAP2
Cytokine Score
Healthy donors
Convalescent
Inflammatory Score
Healthy donors
Convalescent
e
Inflammatory cells
Non-inflammatory cells
HD AE NE
f
S100A12_Score
HD AE NE
g
TLR4_Score
Mono_CD14 78.79%
Mono_C1QA 3.28%
MDSCs 2.34%
Mono_CD14_CD16 15.59%
Others 0.00%
h
CD4_Memory
CD8_Pro
Mono_CD14
significant p = 0.05
false true
i
S100A12_Scores
TLR4_Scores
MYD88_Scores
HD MI SE

Supplementary Fig 4. Details of hyper-inflammatory subtypes associated with cytokine storm in PBMCs, related to Figure 3

a. Bar plots showing the cytokine score and inflammatory score across conditions.

b. UMAP plots of PBMCs colored by: major cell types (Top left panel), inflammatory cell type (Top right panel), inflammatory score (Middle panel) and cytokine score (Bottom panel).

c. Bar plots showing the cytokine score (Top panel) and inflammatory score (Bottom panel) for each cell type.

d. Box plots showing cytokine score in selected cell types.

e. Bar plots showing the proportion of inflammatory cell types (Left panel) and other cell types (Right panel) across conditions.

f. Bar plots showing *S100A12* scores across conditions.

g. Pie charts showing the relative percentage contribution of each cell type to the *TLR4*-score.

h. Dot plot showing the interactions among inflammatory cell types in COVID-19 patients with acute necrotizing encephalopathy. P values are indicated by the circle sizes, as shown in the scale on the right.

i. Box plots showing the expression of selected genes across conditions.

Significant differences in a, d, e, f and i were determined by Two-sided Student's T-test with Bonferroni correction (*$p<0.05$, **$p<0.01$, ***$p<0.001$, ****$p<0.0001$, $^{ns}p>0.05$). The error bars represent Standard Error (SE), and the median is shown as horizontal bars.

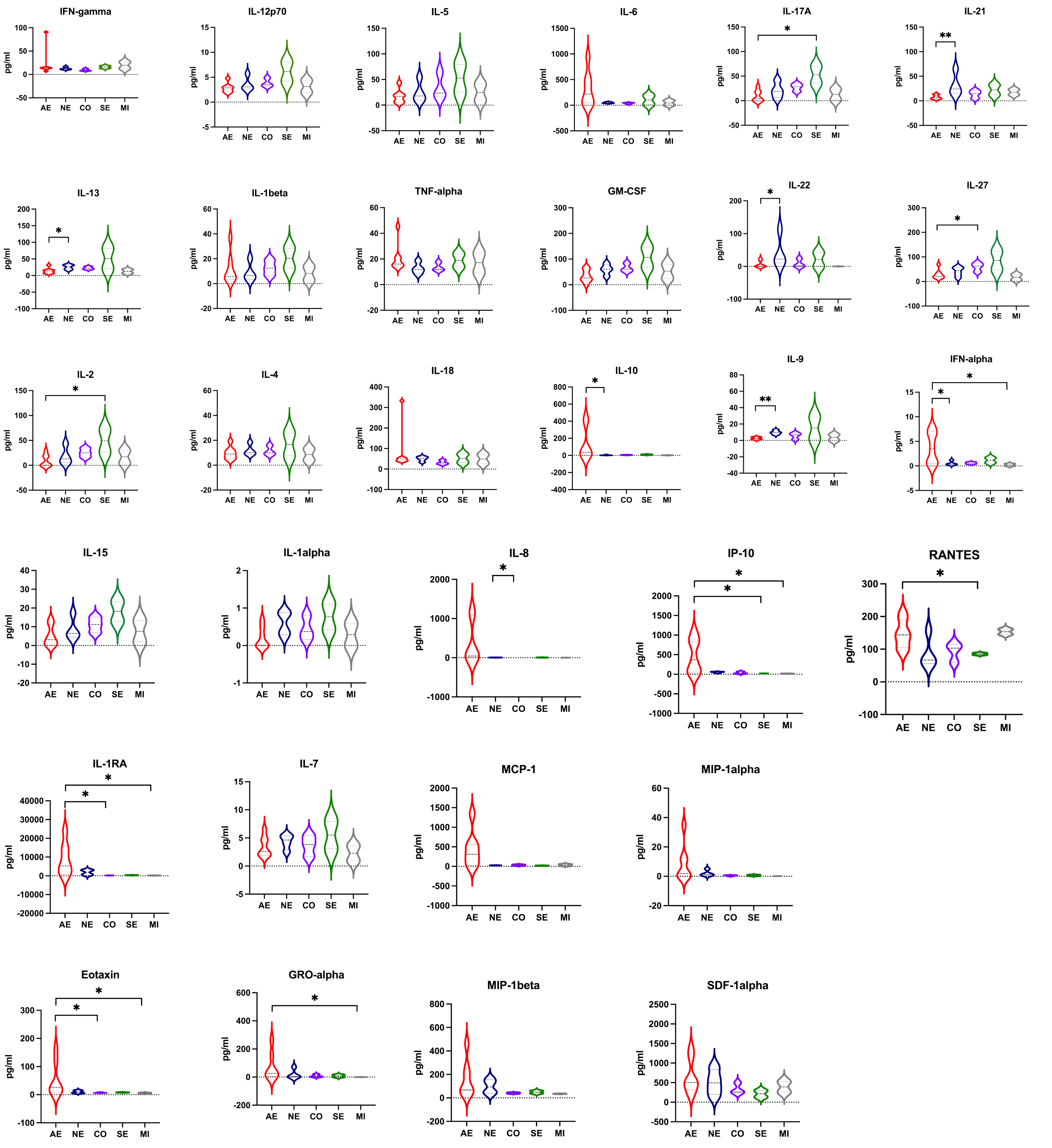

IFN-gamma
IL-12p70
IL-5
IL-6
IL-17A
IL-21
IL-13
IL-1beta
TNF-alpha
GM-CSF
IL-22
IL-27
IL-2
IL-4
IL-18
IL-10
IL-9
IFN-alpha
IL-15
IL-1alpha
IL-8
IP-10
RANTES
IL-1RA
IL-7
MCP-1
MIP-1alpha
Eotaxin
GRO-alpha
MIP-1beta
SDF-1alpha
pg/ml
AE NE CO SE MI

Supplementary Fig 5. The levels of 32 cytokines in each group.

Significant differences were determined by Two-sided Student's T-test with Bonferroni correction (*p<0.05, **p<0.01, ***p<0.001, ****p<0.0001, ^{ns}p>0.05).

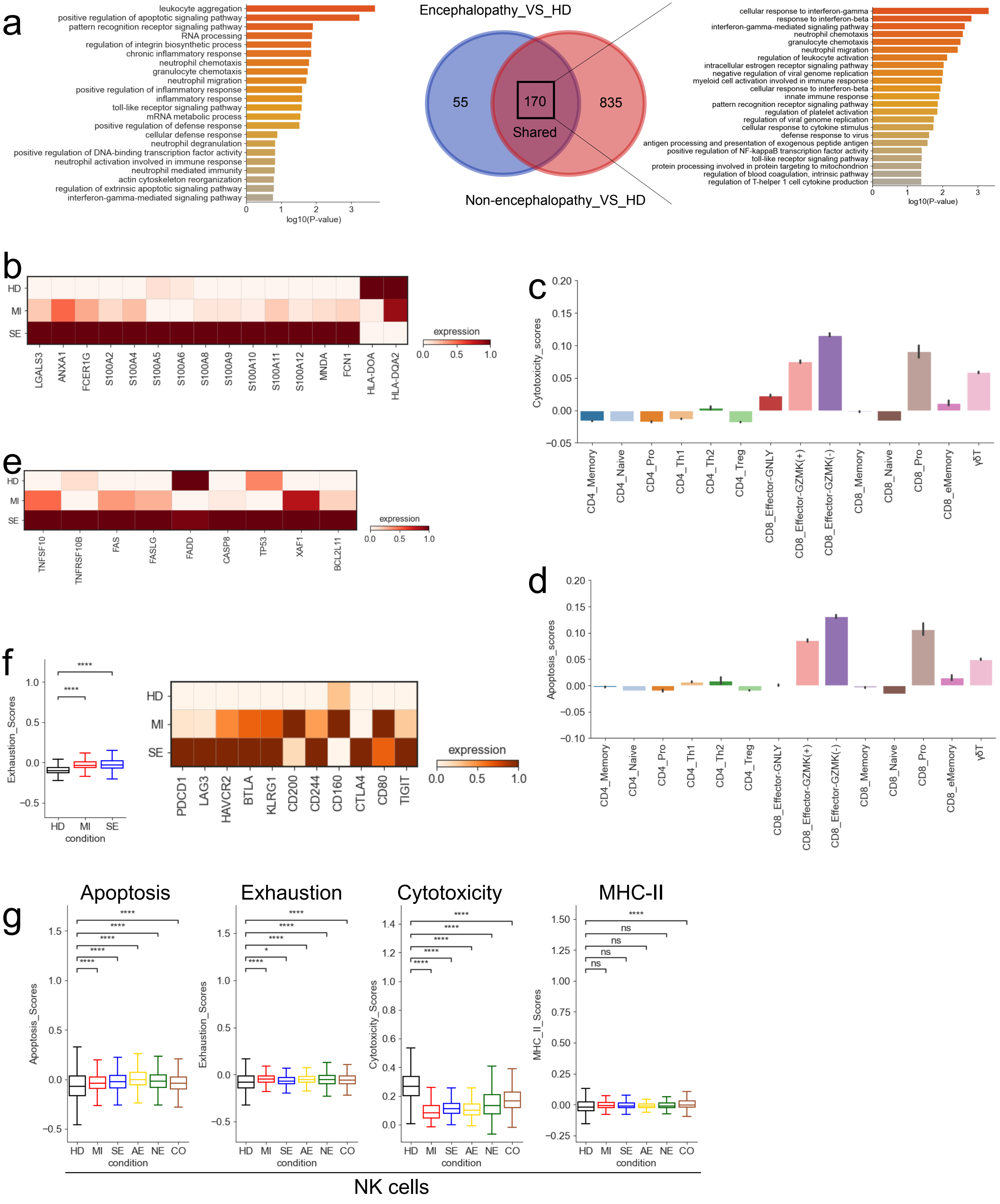

a
leukocyte aggregation
positive regulation of apoptotic signaling pathway
pattern recognition receptor signaling pathway
RNA processing
regulation of integrin biosynthetic process
chronic inflammatory response
neutrophil chemotaxis
granulocyte chemotaxis
neutrophil migration
positive regulation of inflammatory response
inflammatory response
toll-like receptor signaling pathway
mRNA metabolic process
positive regulation of defense response
cellular defense response
neutrophil degranulation
positive regulation of DNA-binding transcription factor activity
neutrophil activation involved in immune response
neutrophil mediated immunity
actin cytoskeleton reorganization
regulation of extrinsic apoptotic signaling pathway
interferon-gamma-mediated signaling pathway
log10(P-value)
Encephalopathy_VS_HD
55
170
Shared
835
Non-encephalopathy_VS_HD
cellular response to interferon-gamma
response to interferon-beta
interferon-gamma-mediated signaling pathway
neutrophil chemotaxis
granulocyte chemotaxis
neutrophil migration
regulation of leukocyte activation
intracellular estrogen receptor signaling pathway
negative regulation of viral genome replication
myeloid cell activation involved in immune response
cellular response to interferon-beta
innate immune response
pattern recognition receptor signaling pathway
regulation of platelet activation
regulation of viral genome replication
cellular response to cytokine stimulus
defense response to virus
antigen processing and presentation of exogenous peptide antigen
positive regulation of NF-kappaB transcription factor activity
toll-like receptor signaling pathway
protein processing involved in protein targeting to mitochondrion
regulation of blood coagulation, intrinsic pathway
regulation of T-helper 1 cell cytokine production
log10(P-value)
b
HD
MI
SE
LGALS3 ANXA1 FCER1G S100A2 S100A4 S100A5 S100A6 S100A8 S100A9 S100A10 S100A11 S100A12 MNDA FCN1 HLA-DOA HLA-DQA2
expression
c
Cytotoxicity_scores
CD4_Memory CD4_Naive CD4_Pro CD4_Th1 CD4_Th2 CD4_Treg CD8_Effector-GNLY CD8_Effector-GZMK(+) CD8_Effector-GZMK(-) CD8_Memory CD8_Naive CD8_Pro CD8_eMemory γδT
e
TNFSF10 TNFRSF10B FAS FASLG FADD CASP8 TP53 XAF1 BCL2L11
d
Apoptosis_scores
f
Exhaustion_Scores
condition
PDCD1 LAG3 HAVCR2 BTLA KLRG1 CD200 CD244 CD160 CTLA4 CD80 TIGIT
g
Apoptosis
Exhaustion
Cytotoxicity
MHC-II
Apoptosis_Scores
Exhaustion_Scores
Cytotoxicity_Scores
MHC_II_Scores
HD MI SE AE NE CO
condition
NK cells

Supplementary Fig 6. Characterization of gene expression differences in T cells across conditions, related to Figure 4

a. GO enrichment analysis of shared DEGs in COVID-19 patients identified by comparing healthy donors and COVID-19 patients with different symptoms (**Left panel**). Venn diagram and GO analysis shows the number of upregulated DEGs of encephalopathy patients and non-encephalopathy patients in T cells, comparisons as indicated (**Right panel**). DEGs refer to genes with Wilcoxon adjusted p value ≤ 0.05.

b. Heatmap of normalized expression for selected genes (neutrophil activation-associated genes and HLA-II molecules) in T cells across different conditions (HD, MI and SE).

c. Bar plots of the cytotoxicity scores in each T cell subtype.

d. Bar plots of the apoptosis scores in each T cell subtype.

e. Heatmap of normalized expression for selected genes (apoptosis-associated genes) in T cells across different conditions (HD, MI and SE).

f. Box plot of exhaustion scores (Left) in T cells across different conditions (HD, MI and SE). Heatmap of normalized expression for selected genes (exhaustion-associated genes) in T cells across different conditions (HD, MI and SE).

g. Box plots of apoptosis, exhaustion, cytotoxicity and HLA-II scores in NK cells across different conditions (HD, MI, SE, NE, AE and CO).

Significant differences in f and g were determined by Two-sided Student's T-test with Bonferroni correction (*$p<0.05$, **$p<0.01$, ***$p<0.001$, ****$p<0.0001$, $^{ns}p>0.05$). The error bars represent Standard Error (SE), and the median is shown as horizontal bars.

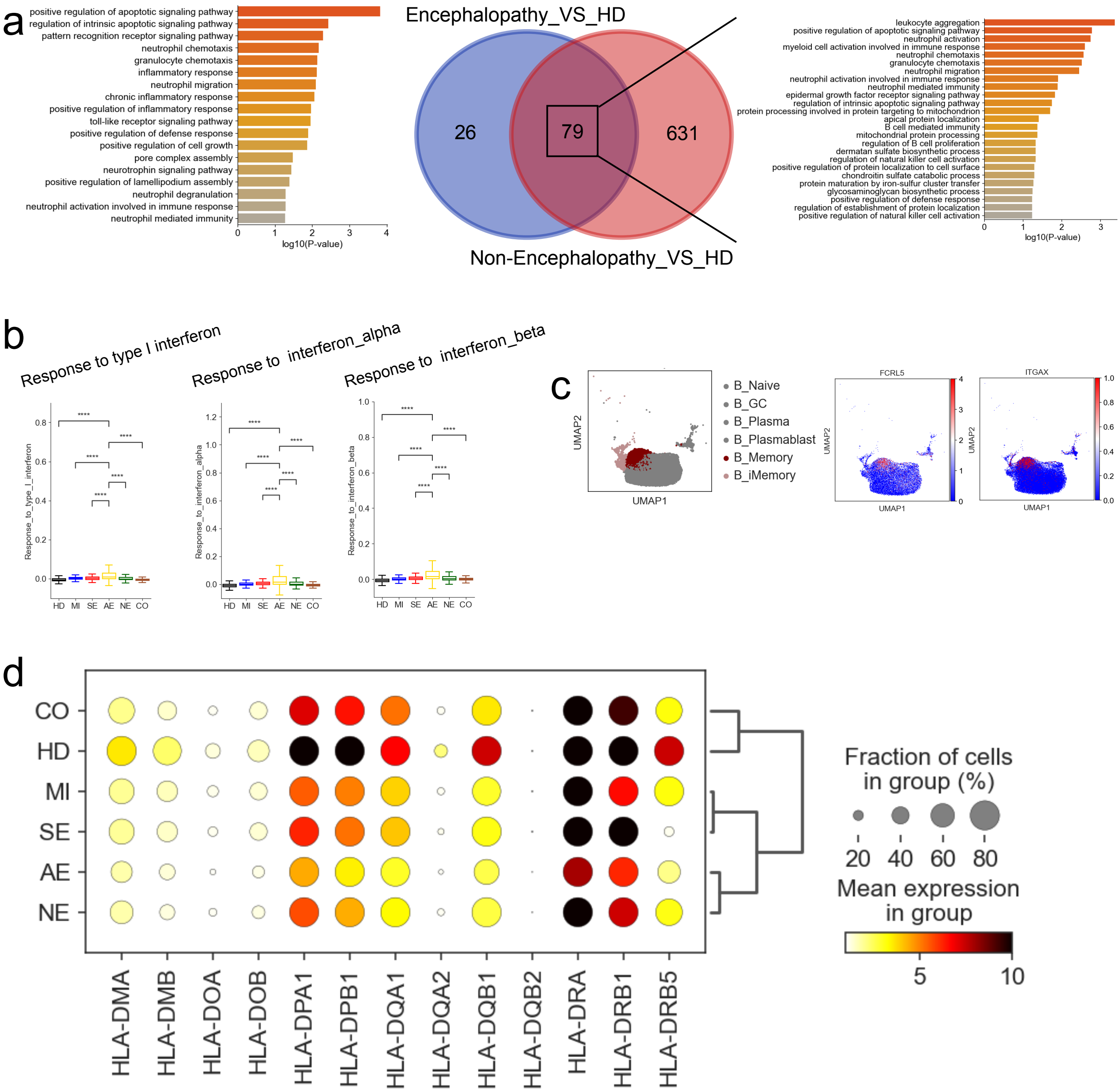
a
positive regulation of apoptotic signaling pathway
regulation of intrinsic apoptotic signaling pathway
pattern recognition receptor signaling pathway
neutrophil chemotaxis
granulocyte chemotaxis
inflammatory response
neutrophil migration
chronic inflammatory response
positive regulation of inflammatory response
toll-like receptor signaling pathway
positive regulation of defense response
positive regulation of cell growth
pore complex assembly
neurotrophin signaling pathway
positive regulation of lamellipodium assembly
neutrophil degranulation
neutrophil activation involved in immune response
neutrophil mediated immunity
log10(P-value)
Encephalopathy_VS_HD
26
79
631
Non-Encephalopathy_VS_HD
leukocyte aggregation
positive regulation of apoptotic signaling pathway
neutrophil activation
myeloid cell activation involved in immune response
neutrophil chemotaxis
granulocyte chemotaxis
neutrophil migration
neutrophil activation involved in immune response
neutrophil mediated immunity
epidermal growth factor receptor signaling pathway
regulation of intrinsic apoptotic signaling pathway
protein processing involved in protein targeting to mitochondrion
apical protein localization
B cell mediated immunity
mitochondrial protein processing
regulation of B cell proliferation
dermatan sulfate biosynthetic process
regulation of natural killer cell activation
positive regulation of protein localization to cell surface
chondroitin sulfate catabolic process
protein maturation by iron-sulfur cluster transfer
glycosaminoglycan biosynthetic process
positive regulation of defense response
regulation of establishment of protein localization
positive regulation of natural killer cell activation
log10(P-value)
b
Response to type I interferon
Response to interferon_alpha
Response to interferon_beta
Response_to_type_I_interferon
Response_to_interferon_alpha
Response_to_interferon_beta
HD MI SE AE NE CO
c
UMAP2
UMAP1
B_Naive
B_GC
B_Plasma
B_Plasmablast
B_Memory
B_iMemory
FCRL5
ITGAX
d
CO
HD
MI
SE
AE
NE
HLA-DMA
HLA-DMB
HLA-DOA
HLA-DOB
HLA-DPA1
HLA-DPB1
HLA-DQA1
HLA-DQA2
HLA-DQB1
HLA-DQB2
HLA-DRA
HLA-DRB1
HLA-DRB5
Fraction of cells in group (%)
20 40 60 80
Mean expression in group
5 10

Supplementary Fig 7. Characterization of gene expression differences in B cells across conditions, related to Figure 5

a. GO enrichment analysis of shared DEGs in COVID-19 patients identified by comparing healthy donors and COVID-19 patients with different symptoms (**Left panel**). Venn diagram and GO analysis shows the number of upregulated DEGs of encephalopathy patients and non-encephalopathy patients in B cells, comparisons as indicated (**Right panel**). DEGs refer to genes with Wilcoxon adjusted p value ≤0.05.

b. Box plots of three GO terms in B cells across different conditions. Y axis represents the normalized expression score of gene sets related to response_to_type_I_interferon (GO: 0034340), response_to_interferon_alpha (GO: 0035455) and response_to_interferon_beta (GO: 0035456), colored by condition.

c. UMAP plots of B cells colored by: major cell types (Left panel), FCRL5 score (Middle panel), and ITGAX score (Right panel).

d. Dot plots of the selected genes (HLA-II molecules) in B cells across conditions.

Significant differences in b were determined by Two-sided Student's T-test with Bonferroni correction (*p<0.05, **p<0.01, ***p<0.001, ****p<0.0001, ^{ns}p>0.05). The error bars represent Standard Error (SE), and the median is shown as horizontal bars.

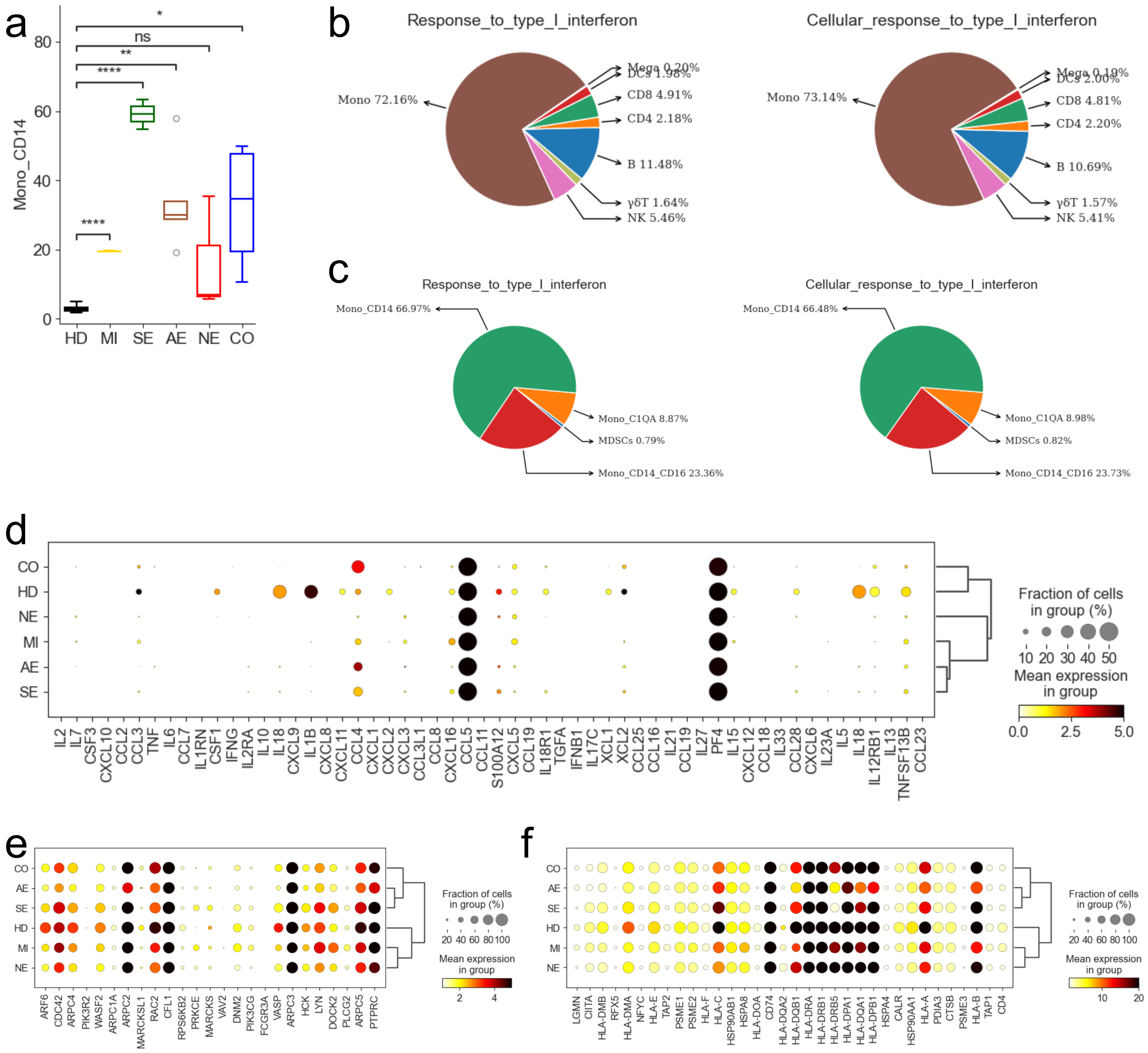
a
b
c
d
e
f
Mono_CD14
HD MI SE AE NE CO
Response_to_type_I_interferon
Cellular_response_to_type_I_interferon
Mono 72.16%
Mega 0.20%
DCs 1.98%
CD8 4.91%
CD4 2.18%
B 11.48%
γδT 1.64%
NK 5.46%
Mono 73.14%
Mega 0.19%
DCs 2.00%
CD8 4.81%
CD4 2.20%
B 10.69%
γδT 1.57%
NK 5.41%
Mono_CD14 66.97%
Mono_C1QA 8.87%
MDSCs 0.79%
Mono_CD14_CD16 23.36%
Mono_CD14 66.48%
Mono_C1QA 8.98%
MDSCs 0.82%
Mono_CD14_CD16 23.73%
Fraction of cells in group (%)
Mean expression in group

Supplementary Fig 8. Characterization of gene expression differences in myeloid cells across conditions, related to Figure 6

a. Box plot showing the relative proportion of Mono_CD14 across HD, MI, SE, NE, AE and CO conditions.

b. Pie charts showing the relative percentage contribution of each major cell type to the Resposne_to_type_I_interferon (GO: 0034340) and cellular_response_to_type_I_interferon (GO: 0071357).

c. Pie charts showing the relative percentage contribution of each monocyte subtype to the resposne_to_type_I_interferon (GO: 0034340) and cellular_response_to_type_I_interferon (GO: 0071357).

d. Dot plots of selected genes (inflammatory-related genes) in megakaryocytes across conditions.

e. Dot plots of selected genes (phagocytosis-related genes) in DCs across conditions.

f. Dot plots of selected genes (antigen presentation-related genes) in DCs across conditions.

Significant differences were determined by Two-sided Student's T-test with Bonferroni correction (*p<0.05, **p<0.01, ***p<0.001, ****p<0.0001, ^{ns}p>0.05). The error bars represent Standard Error (SE), and the median is shown as horizontal bars.
