# Supplementary material for: Single-cell transcriptomic analysis reveals a systemic immune dysregulation in COVID-19-associated pediatric encephalopathy
Source: Signal Transduct Target Ther. 2023 Oct 18;8:398. doi: 10.1038/s41392-023-01641-y (PMC10582072; doi:10.1038/s41392-023-01641-y)
Supplement: Supplementary file 2 — Supplementary Table 1 [file 41392_2023_1641_MOESM2_ESM.docx]

Supplementary Table 1 (Sheet 1). Clinical features and laboratory findings of COVID-19 patients

| Acute necrotizing encephalopathy | | | | | |
| --- | --- | --- | --- | --- | --- |
| Brain MRI | 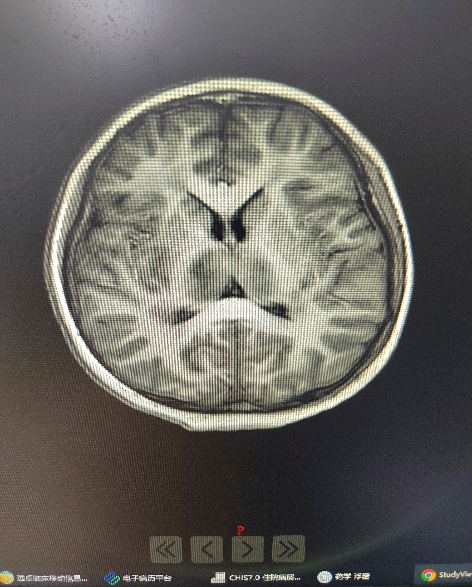 | \| None \| \| --- \| \|  \| \|  \| \|  \| | 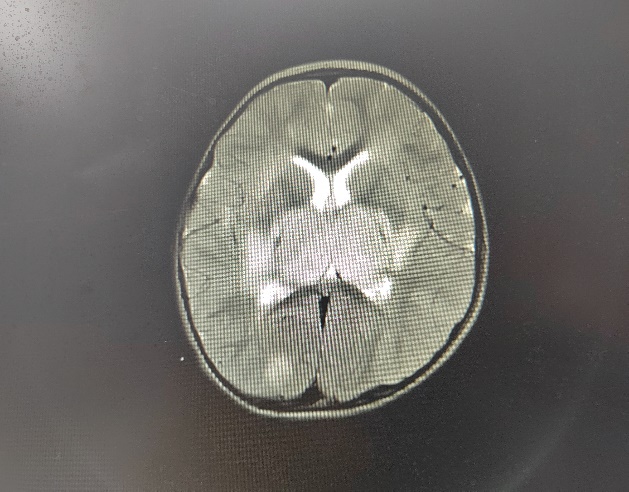 | 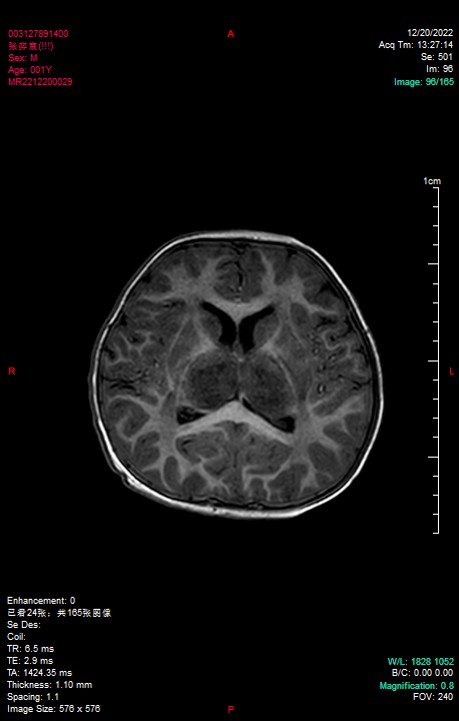 | 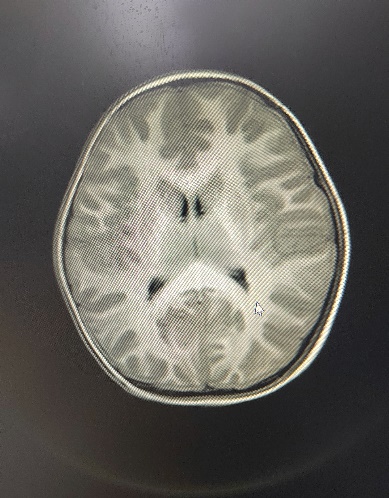 |
| Code | AE1 | AE1_1 | AE2 | AE3 | AE4 |
| Gender | Male | Male | Female | Male | Male |
| Age (years) | 9y1m | 9y1m | 1y10m | 1y5m | 3y |
| Vaccination history | Two shots of vaccine | Two shots of vaccine | Unvaccinated | Unvaccinated | Unvaccinated |
| SARS-CoV-2 PCR (Genotype) | Positive (BA.5.2) | Positive (BA.5.2) | Positive (BF.7) | Positive (BF.7) | Positive (BA.5.2) |
| Clinical features | Fever, unconsciousness | Fever, unconsciousness | Fever, hyperspasmia, unconsciousness | Fever, hyperspasmia, unconsciousness | Fever, hyperspasmia, unconsciousness |
| Brain MRI | Bilateral symmetrical thalamus, cerebellum, dorsal pontine, midbrain, and bilateral temporal lobe white matter, putamen, external capsule, paraventricular white matter pathologic signal patterns | None | bilateral diffuse thalamus swelling, bilateral symmetrical cerebral white matter, cerebellar and dorsal brain stem pathologic signal patterns | bilateral diffuse thalamus and cerebellum swelling, bilateral symmetrical thalamus ,cerebella rdentate nucleus，centrum semiovale and dorsal brain stem pathologic signal patterns | bilateral diffuse thalamus and brain stem swelling, bilateral cerebral white matter multiple pathologic signal patterns |
| Clinical outcome | Extubated on day 8 and discharged on day 14 | Extubated on day 8 and discharged on day 14 | Death 6 days after withdrawal of life support | Died on the 29h of admission after withdrawal of life support | Died on the 14h of admission |
| Non-acute necrotizing encephalopathy | | | | | |
| Brain MRI | \| 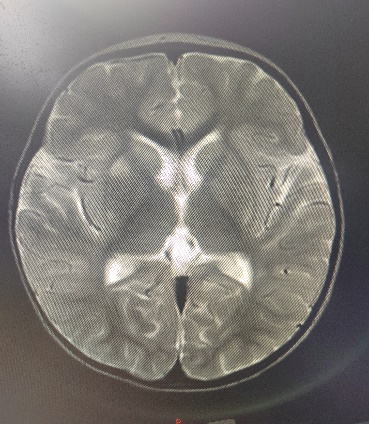 \| \| --- \| \|  \| \|  \| \|  \| \|  \| \|  \| \|  \| | 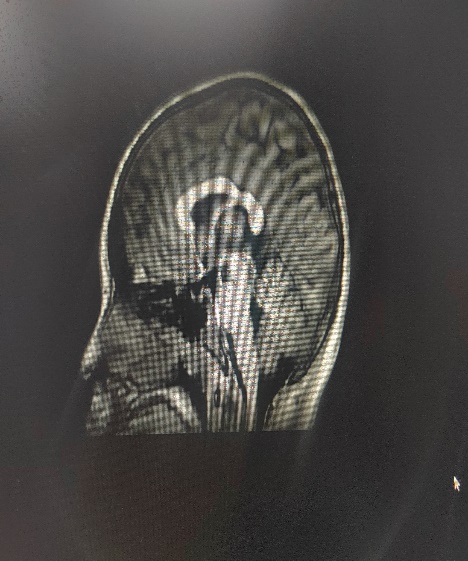 | 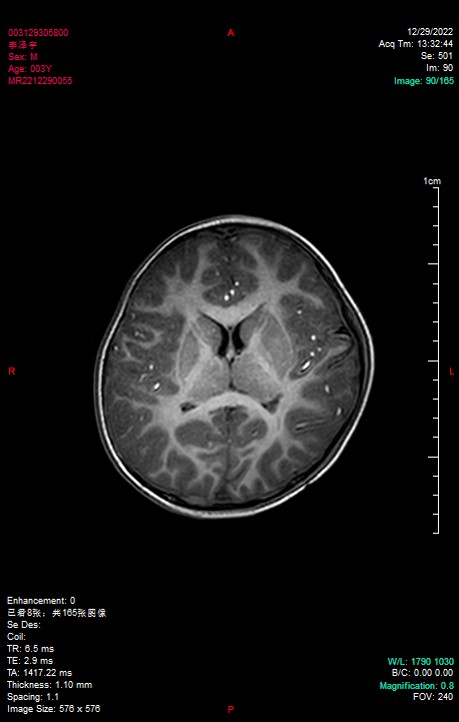 |  |  |
| Code | NE1 | NE2 | NE3 |  |  |
| Gender | Male | Male | Male |  |  |
| Age (years) | 1y2m | 10y8m | 3y |  |  |
| Vaccination history | Unvaccinated | Two shots of vaccine | Unvaccinated |  |  |
| SARS-CoV-2 PCR (Genotype) | Positive (BA.5.2) | Positive (BF.7.) | Positive (BF.7.) |  |  |
| Clinical features | Fever, drowsiness, unconsciousness | Fever, unconsciousness | Fever, hyperspasmia, unconsciousness |  |  |
| Brain MRI | Abnormal intensities in the head of right caudate nucleus and bilateral lenticular nucleus on FLAIR, T1, and T2-weighted images | Day1 MRI: Abnormal intensities in the splenium of the corpus callosum and brain stem. Abnormal signals in the vast portion of the corpus callosum in DWI.  D7 MRI: normal | Bilateral cerebral swelling, mainly in cortex and subcortical white matter, with diffuse and extensive DWI high signal. DWI revealed bilateral subcortical white matter predominance with bright tree appearance |  |  |
| Clinical outcome | Discharged on day 6 | Discharged on day 9 | Extubated on day 7 and discharged on day 15 |  |  |
| Mild | | | Severe | | |
| X-ray or CT | 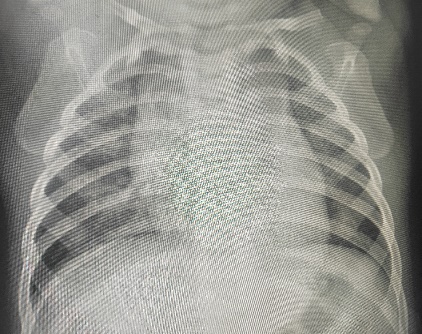 | 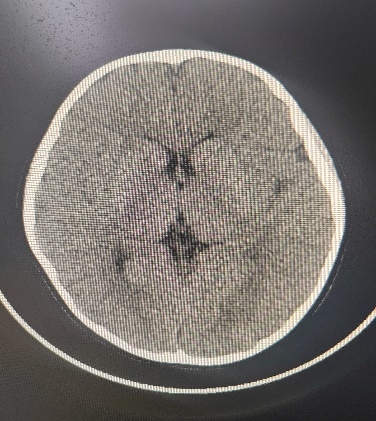 | 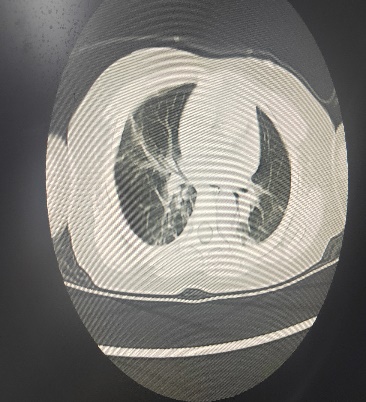 | 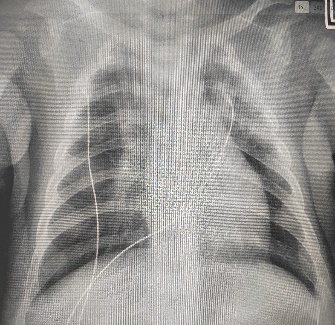 |  |
| Code | MI1 | MI2 | SE1 | SE2 |  |
| Gender | Male | Female | Male | Male |  |
| Age (years) | 8m13d | 3y | 2y11m | 5m15d |  |
| Vaccination history | Unvaccinated | Unvaccinated | Unvaccinated | Unvaccinated |  |
| SARS-CoV-2 PCR (genotype) | Positive (--) | Positive (--) | Positive (BF.7) | Positive (--) |  |
| Clinical features | Fever, cough, trachyphonia, shortness of breath | Fever, cough,  hyperspasmia | Fever, drowsiness,  trachyphonia, shortness of breath | Fever, cough, pant, dyspnea |  |
| Diagnosis | Laryngitis, laryngeal obstruction (Grade II) | Acute upper respiratory infection, febrile convulsion | Laryngitis, laryngeal obstruction (Grade III) | Laryngitis, laryngeal obstruction (Grade III), Severe bronchopneumonia |  |
| Clinical outcome | Improvement | Improvement | Extubated on day 2 and discharged on day 4 | Extubated on day 9 and discharged on day 14 |  |

Supplementary Table 1 (Sheet 2). Additional demographic characteristics of healthy donors

| **Participants ID ^a^** | **Condition** | **Sex ^b^** | **Age (year)** | **ScRNA-seq** | **Bacterial or viral infections within 6 months** | **Sample type** |
| --- | --- | --- | --- | --- | --- | --- |
| HD1 | HD | 1 | 8 | Yes | No | Fresh PBMC |
| HD2 | HD | 1 | 8 | Yes | No | Fresh PBMC |
| HD3 | HD | 1 | 8 | Yes | No | Fresh PBMC |
| HD4 | HD | 2 | 8 | Yes | No | Fresh PBMC |
| HD5 | HD | 2 | 7 | Yes | No | Fresh PBMC |
| HD6 | HD | 2 | 8 | Yes | No | Fresh PBMC |

^a^ Participants ID, the ID of a participants.

b Sex, 1, Male; 2, Female.
